# The RNA processing factors THRAP3 and BCLAF1 promote the DNA damage response through selective mRNA splicing and nuclear export

**DOI:** 10.1093/nar/gkx1046

**Published:** 2017-11-03

**Authors:** Jekaterina Vohhodina, Eliana M. Barros, Abigail L. Savage, Fabio G. Liberante, Lorenzo Manti, Peter Bankhead, Nicola Cosgrove, Angelina F. Madden, D. Paul Harkin, Kienan I. Savage

**Affiliations:** 1 Centre for Cancer Research and Cell Biology, Queen’s University Belfast, 97 Lisburn Rd, Belfast BT9 7BL, UK; 2 Dipartimento di Fisica ‘E Pancini’, Università di Napoli Federico II, Monte S. Angelo, 80126 Napoli, Italy; 3 Endocrine Oncology Research Group, Department of Surgery, Royal College of Surgeons in Ireland, Dublin 2, D02 YN77, Ireland

## Abstract

mRNA splicing and export plays a key role in the regulation of gene expression, with recent evidence suggesting an additional layer of regulation of gene expression and cellular function through the selective splicing and export of genes within specific pathways. Here we describe a role for the RNA processing factors THRAP3 and BCLAF1 in the regulation of the cellular DNA damage response (DDR) pathway, a key pathway involved in the maintenance of genomic stability and the prevention of oncogenic transformation. We show that loss of THRAP3 and/or BCLAF1 leads to sensitivity to DNA damaging agents, defective DNA repair and genomic instability. Additionally, we demonstrate that this phenotype can be at least partially explained by the role of THRAP3 and BCLAF1 in the selective mRNA splicing and export of transcripts encoding key DDR proteins, including the ATM kinase. Moreover, we show that cancer associated mutations within THRAP3 result in deregulated processing of THRAP3/BCLAF1-regulated transcripts and consequently defective DNA repair. Taken together, these results suggest that THRAP3 and BCLAF1 mutant tumors may be promising targets for DNA damaging chemotherapy.

## INTRODUCTION

RNA processing and its further translation are compartmentalized processes occurring in the nucleus and cytoplasm respectively. Pre-mRNA synthesis and maturation occurs through a multi-step process involving transcription, addition of a 5′ methyl-guanine (m^7^G) cap, removal of intronic sequences by splicing and cleavage of the 3′ end followed by polyadenylation (1), thus leading to the production of a stable mature RNA and facilitating its export from the nucleus for further translation (2,3). Each step involves engagement with different sets of RNA-binding proteins (RBPs), hnRNPs and SRs, which co-ordinate to yield a mature mRNA and transport it to the ribosome for translation and subsequent protein expression (4,5). Moreover, additional layers of complexity are introduced through the mechanistic coupling of transcription to capping, 3′ end processing to splicing and splicing to export. For example, the mammalian capping enzyme (comprised of RNA 5′-triphosphatase, guanylyl-transferase and (guanine-N7) methyltransferase) binds to the phosphorylated C-terminal domain of RNA polymerase II allowing the latter to proceed to the transcription elongation step (6); the nuclear protein ALY is recruited to mRNAs during splicing, through interaction with the spliceosome component UAP56, thus leading to successful mRNA export (7,8). Indeed, the coupling of each successive step within the RNA synthesis/maturation pathway may serve as an additional level of control of gene expression. Recent evidence also suggests multiple levels of selectivity in both mRNA splicing and export with multiple cellular processes controlled by selective splicing and export, including maintenance of pluripotency, stress responses, proliferation, survival/apoptosis, haematopoiesis and DNA repair (9–11). Indeed, a recent study investigating the impact of SR proteins on mRNA splicing found that the serine arginine rich (SR) mRNA-splicing proteins SRSF1–SRSF7, both contribute to the mRNA splicing of distinct groups of genes (with each SRSF protein regulating different pools of genes) and also couple this selective splicing to selective mRNA export, by acting as adaptor proteins allowing the binding of the mRNA export factor NXF1 to target transcripts (11). Additionally, specific genes have also been shown to be regulated at the mRNA splicing and export levels following specific stimuli. In particular, the involvement of RNA splicing and export factors (SFs) within the cellular DNA damage response (DDR) pathway is now becoming evident (12,13).

In keeping with this, a novel BRCA1-interacting RNA splicing complex, containing many members of the core RNA splicing machinery such as BCLAF1, THRAP3 and SF3B1, has been recently identified by our group (14). This complex is formed in response to DNA damage through the ATM-dependent phosphorylation of BRCA1, which is constitutively bound to the promoters of a large subset of genes. Formation of this complex on genetic promoters bound by BRCA1, promotes their pre-mRNA splicing and subsequent transcript stability in response to DNA damage. One particular member of this complex, THRAP3, has been shown to be phosphorylated at five different serine residues, in an ATR kinase-dependent manner, in response to different DNA damaging agents, as well as PARylated in response to oxidative stress, which further facilitates its localization to nuclear speckles along with other splicing factors (15,16). Moreover, THRAP3 depletion sensitizes cells to the replication inhibitor hydroxyurea (HU), suggesting a function for THRAP3 in the DDR (15). Importantly, THRAP3 deficiency has been observed in multiple cancer types, such as breast and ovarian cancers, oral squamous cell carcinomas, liver tumors and parathyroid carcinomas, suggesting that it may function as a tumor suppressor gene, possibly through a role in the DDR (17–19).

Despite the regulation of THRAP3 in response to DNA damage, here we report a new function for THRAP3 and BCLAF1 in RNA splicing and export independent of DNA damage and their role in the BRCA1/BCLAF1 complex. More specifically, our study shows that, together with BCLAF1, THRAP3 selectively regulates the constitutive mRNA splicing and export of a specific subset of transcripts, many of which are involved in the cellular DDR.

Moreover, we demonstrate that BCLAF1 is able to partially compensate for the absence of THRAP3 and *vice versa*, with double knockdown of both factors leading to severe cellular sensitivity to DNA damage and DNA repair deficiency. We also show that this sensitivity to DNA damaging agents can be at least partially explained by the involvement of THRAP3 and BCLAF1 in the processing of transcripts encoding members of the FA/BRCA-related DNA repair pathway, including ATM. Specifically, we found that BCLAF1 promotes the splicing of these transcripts, whilst THRAP3 promotes their export to the cytoplasm, both tightly linked processes required for transcript stability and protein expression. Finally, we show that cancer-associated mutations identified within THRAP3 induce a severe defect in DNA double strand break (DSB) repair capability. Thus, our results suggest that tumors harboring mutations within THRAP3 may be promising targets for DNA damaging therapies.

## MATERIALS AND METHODS

### Cell lines

293T and U2OS cells were obtained from the Cancer Research UK cell Repository (London Research Institute, London). U2OS-DR-GFP cells were a kind gift from Prof Jeremy Stark (Beckman Research Institute of the City of Hope, Duarte, CA, USA). All cell lines were maintained in Dulbecco’s modified Eagle’s medium media (Sigma-Aldrich) containing 10% fetal calf serum. Additionally, all cells used in this study were thawed directly from short tandem repeat (STR) profiled stocks to ensure their validity.

### siRNAs

siRNAs with the following sequences were obtained from QIAGEN; siBRCA1: 5′- ACC ATA CAG CTT CAT AAATAA -3′, siBCLAF1_2: 5′CUA GAU UAC UUC AGU GAU ATT-3′ 5′, siBCLAF_4 5′-UAG UAG AGA UCG UAUGUA UTT-3′ (siBCLAF1 contains 50% siBCLAF1_2 and 50% siBCLAF_4 siRNAs). siTHRAP3_7 5′- ACGAGTTGAATCATTGTTCAA -3′, siTHRAP3_8 5′- CCCGTTCTTCCTCCAACCATA -3′ (siTHRAP3 contains 50% siTHRAP3_7 and 50% siTHRAP3_8 siRNAs). siTHRAP3+siBCLAF1 contains 50% siBCLAF1 2/4 and 50% siTHRAP3 7/8 siRNAs. siRNA oligos were delivered to a final concentration of 10nM by reverse transfection using RNAiMax (Invitrogen) according to manufacturer's instructions with cells incubated for 48–72 h following transfection (dependent on the length of the assay being carried out).

### Plasmids

pcDNA3.1(+)-THRAP3-FLAG, pcDNA3.1 (+)-THRAP3-ΔN190-FLAG, pcDNA3.1 (+)-THRAP3-ΔC359-FLAG and pcDNA3.1 (+)-THRAP3-ΔNC-FLAG plasmids were kindly provided by professor Woan-Yuh Tarn, National Taiwan University, Taipei, Taiwan (20). The pRFP2GFP-ATM construct was generated using the pmaxGFP vector as a backbone. The RFP (red fluorescent protein) sequence was amplified by polymerase chain reaction (PCR) from pmTagRFP-T2-N1 (Michael Davidson Lab via Addgene) using the following primer sequences: Forward 5′-ATGGTGTCTAAGGGCGAAGAG-3′ and Reverse 5′-GCGGTACCGTCGACTGCA-3′ plus 20 bp of homology and HindIII and SalI sites in the reverse primer. This PCR product was inserted into the pmaxGFP vector upstream of the turbo-GFP (green fluorescent protein) open reading frame (ORF) between the KpnI and AgeI sites using Gibson assembly (NEB). The sequence encoding the ATM exon20-intron20-exon21 was PCR amplified from 293T derived genomic DNA using the following primer sequences: Forward 5′- GTGAAGCTTTCCAATGTGTGTTCTTTGTATC-3′ and Reverse 5′- TACGTCGACCTCAAGCAAAGTTTTAAG-3′, and cloned into the pRFP2GFP vector’s ORF between RFP and GFP tags using novel HindIII and SalI sites. pCMV6-AN-Myc-DDK was purchased from Origene (PS100016). The ORF of THRAP3 was inserted in this plasmid using SgfI and MluI sites. THRAP3, cloned in this vector, was expressed as the N-terminal Myc-DDK-tagged protein.

### Site directed mutagenesis

The QuickChange Site Directed Mutagenesis kit (Stratagene) was used, as per the manufacturer's instructions, to introduce the C301>T and C2509>T substitutions within the N-Myc-DDK-THRAP3 construct using the following primers: THRAP3 C301T Forward 5′- CATGGGGCCAATATAACTGAGGAGGCTATGGAAAC -3′, THRAP3 C301T Reverse 5′- GTTTCCATAGCCTCCTCAGTTATATTGGCCCCATG -3′, THRAP3 C2509T Forward 5′- GAACCT TTCAGTTTTGAGCCAGAGGAAGAG -3′, THRAP3 C2509T Reverse 5′- CTCTTCCTCTGGCTCAAAACTGAAAGGTTC -3′

### Nuclear and cytoplasmic RNA fractionation

Cells were trypsinized and centrifuged at 300 *g* for 5 min. The plasma membrane was then lysed with 175 μl of RLN buffer (50 mM Tris–HCl, pH 8.0, 140 mM NaCl, 1.5 mM MgCl_2_, 0.5% (v/v) Nonidet P-40 (1.06 g/ml)) for 5 min on ice. Lysate was then centrifuged for 2 min at 300 *g*. The supernatant, containing cytoplasmic RNA, was transferred to a new tube, whereas the remaining pellet was used for obtaining nuclear RNA which was extracted using Qiagen RNeasy mini kit as per the manufacturer’s instructions. The purity of cytoplasmic and nuclear RNA fractions was always checked by assessing the expression of the nuclear specific RNA *MALAT1* via quantitative reverse transcriptase (qRT)-PCR.

### qRT-PCR and splicing analysis

A total of 1 μg of DNAse (Invitrogen) treated RNA was used for cDNA synthesis using the Transcriptor High Fidelity cDNA Synthesis Kit (Roche Applied Science) according to the manufacturer’s instructions. qRT-PCR was performed using primers specific to each transcript or to *ACTB* mRNA, on both RT positive and RT negative generated cDNA. All reciprocal qRT-PCR reactions performed on RT−ve cDNA were assessed for the absence of any cDNA amplification indicating no genomic DNA contamination prior to further analysis of data. All qRT-PCR reactions were carried out on a ROCHE LightCycler 480 using SYBR Green 480 I Master Mix (ROCHE) as per the manufacturer’s instructions. mRNA concentration levels were then assessed using the ROCHE Relative Quantification algorithm, utilizing in-run standard curve qRT-PCR data generated for each primer set from a serially diluted RNA standard. For quantitative splicing analysis, introns/exons were chosen for assessment in splicing analysis based on their suitability for optimal qRT-PCR primer design. Primers spanning exon-exon boundaries (post-spliced) and exon-intron boundaries (pre-spliced) were designed using the ROCHE universal probe library and manually inputting exon-exon and exon–intron flanking sequences. Using these primers (listed below) levels of pre-spliced and post-spliced mRNAs were assessed as above and normalized to ACTB expression levels within the same samples. Primers used for qRT-PCR analysis were as follows: MALAT1 Forward: 5′-GACGGAGGTTGAGATGAAGC-3′, MALAT1 Reverse: 5′-ATTCGGGGCTCTGTAGTCCT-3′, ACTB Forward: 5′-AGACCTGTACGCCAACACAG-3′, ACTB Reverse: 5′-GGAGCAATGATCTTGATCTTCA-3′, THRAP3 Forward: 5′-GCCGATCTCTCTCTCGTCA-3′, THRAP3 Reverse: 5′-TTGGGTGGTTTCTTTCTCTGTTA-3′, BCLAF1 Forward: 5′-CGACCACGAGGAACCTTTT-3′, BCLAF1 Reverse: 5′-TTTGTCCCAGCAAAAACTCC-3′, ATM_Exon20_Forward: 5′-TTTCTTACAGTAATTGGAGCATTTTG-3′, ATM_Exon21_Reverse: 5′-GGCAATTTACTAGGGCCATTC-3′, ATM_Intron20_Reverse: 5′-CAAGCGACTGAGGGAAACAC-3′, FANCD2_Exon36_Forward: 5′-CCCAGAACTGATCAACTCTCCT-3′, FANCD2_Exon37_Reverse: 5′-CCATCATCACACGGAAGAAA-3′, FANCD2_Intron37_Reverse: 5′-ACAGGTGTGTGCCACCGTG-3′, BRCA2_Exon18_Forward: 5′-CCTGATGCCTGTACACCTCTT-3′, BRCA2_Exon19_Reverse: 5′-GCAGGCCGAGTCACTGTTAGC-3′, BRCA2_Intron18_Reverse: 5′-TACATCTAAGAAATTGAGCATCCT-3′, FANCL_Exon6_Forward: 5′-TCACTCTCAAGTTGAAGGCAA-3′, FANCL_Exon7_Reverse: 5′-GCTTATTAAGGAGCTCTGAGGAGA-3′, FANCL_Intron6_Reverse: 5′-TGGAAACGTGGTGAATAAATTCT-3′, PALB2_Exon9_Forward: 5′-CCTTTCTGATCAACAAGTAGAAGTCA-3′, PALB2_Exon10_Reverse: 5′-GGGGCATCAAAAATTGGTTT-3′, PALB2_Intron9_Reverse: 5′-AGTGTTGATGCGGTACATGCTTAT-3′, Rad51_Exon6_Forward: 5′-TGAGGGTACCTTTAGGCCAGA-3′, Rad51_Exon7_Reverse: 5′-CACTGCCAGAGAGACCATACC-3′, Rad51_Intron6_Reverse: 5′-AGAGACATTCTTCGGCCAAACT-3′.

### Clonogenic survival assays

293T or U2OS cells were transfected with siRNAs and incubated for 62 h. Cells were then seeded as single cells at various densities. A total of 10 h later cells were mock treated or exposed to IR or medium containing HU, Mitomycin C or olaparib (Sigma-Aldrich). After 7–14 days cells were fixed, stained with crystal violet and colonies counted. Surviving fraction for each treatment/dose was calculated by normalizing to plating efficiency for each siRNA treatment.

### Ionizing radiation (IR)

Irradiations (IR) were carried out using an X-RAD 225 kV X-ray generator (Precision X-ray Inc. Branford, CT, USA) at a dose rate of 0.591 Gy.min-1.

### Western blotting

Whole cell extracts were prepared by lysing cells in two volumes of Lysis Buffer (250 mM NaCl, 5 mM ethylenediaminetetraacetic acid (EDTA), 50 mM HEPES, 0.1% v/v NP40). A total of 60 μg of whole cell extract (WCE) was resolved on 4–12% NOVEX gels using MOPS buffer (Invitrogen). Protein was transferred to PVDF membrane (Invitrogen) and blotted for BCLAF1 (BTF 608A, Bethyl Labs); THRAP3 (A300–956A, Bethyl Labs), GAPDH (ab9485, Abcam), pATM (S1981) (ab79891 Abcam), ATM (2C1, Santa Cruz), pATR (S428) (2853, Cell Signaling), ATR (N-19, Santa Cruz), 53BP1 (NB100–34, Novus Biologicals), γH2AX (S139) (JBW301, Millipore), cyclin B1 (GNS1) (sc-245, Santa Cruz), pP53 (S15) (9284S, Cell Signaling), pKAP1 (S824) (A300–767A, Bethyl Labs), FANCD2 (FI17) (sc200–22, Santa Cruz), BRCA2 (H-300) (sc-8326, Santa Cruz), FANCL (B-11) (sc-137067, Santa Cruz), PALB2 (H-45) (sc-382436, Santa Cruz) and Rad51 (3C10) (sc-53428, Santa Cruz).

### DNA repair assay–immunofluorescence

Cells were transfected with siRNAs and incubated for 24 h. Cells were then plated onto coverslips and incubated for a further 24–48 h (depending on the post-treatment time point required) before treatment with 2Gy IR using an XRAD 225 X-ray generator (Precision X-ray Inc. Branford, CT, USA) at a dose rate of 0.591 Gy.min-1. Cells were fixed at 0, 1 and 24 h post-IR in 4% paraformaldehyde/phosphate buffered saline (PBS) and then permeabilized in 0.2% Triton X-100/PBS followed by blocking in 3% BSA/PBS. Cells were then stained with anti-γ-H2AX/53BP1 primary antibody and anti-mouse AlexaFluor 488/568 (Invitrogen) secondary antibody. Cells were also counterstained with DAPI (4′,6-Diamidine-2′-phenylindole dihydrochloride)to identify cellular nuclei. Cells containing more than 5 γ-H2AX/53BP1 foci were scored and representative images acquired using a Nikon Eclipse Ti-S fluorescence microscope, with 60× objective.

### RNA–FISH

Cells were transiently transfected with siRNA (siCtrl, siTHRAP3, siBCLAF1, siTHRAP3+siBCLAF1) and left for 48 h. Cells were further seeded onto glass coverslips at 80% confluency and 24 h later were fixed with 4% paraformaldehyde in PBS for 5 min at room temperature, washed with 1× PBS for 5 min, and then left overnight in 100% ethanol at −20°C. The next day cells were permeabilized for 10 min with 1× PBS containing 0.5% Triton X-100 and washed with 1× PBS. Pre-hybridization step was carried out for 15 min at 37°C in pre-hybridization buffer (2× SSC, 20% formamide, 0.2% BSA, 1 mg/ml yeast tRNA (Sigma)). Cells were further incubated in a humidified chamber (in the dark) at 37°C for 3 h in hybridization buffer (pre-hybridization buffer + 10% dextran sulphate) with oligo dT primer, after which cells were washed 2× for 5 min in: 2× SSC/ 20% formamide at 42°C, 2× SSC at 42°C, 1× SSC at RT, 1× PBS at RT. Coverslips were mounted with ProLong Gold with DAPI. ATM specific probe was left for incubation over night at 42°C. The rest of the procedure was performed as described above. The sequences of the probes used were PolyA-fluorescence *in situ* hybridization (FISH) Cy3,5′TTTTTTTTTTTTTTTTTTTTTTTTTTTTTTTTTTTTTTTTTTTTTTTTTT3′, ATM-FISH Cy3,5′AAAATGGGAATAGAGCAAAATATGTGTGAAGTAAATAGAACTTTTCTTT3′.

Cells were imaged using a Nikon-TiS fluorescence microscope and for quantitative comparison of fluorescent signals the image acquisition parameters were maintained for all acquisitions. The nuclear/cytoplasmic ratio of mRNA–FISH fluorescent signals was measured using an in-house developed ImageJ plugin/algorithm designed to identify cellular nuclei from the DAPI channel of a two channel image (channel 1 being DAPI and channel 2 being our fluorescent RNA–FISH probe signal) and apply this as a mask on the channel-imaged for fluorescent RNA–FISH probes (21). The mean nuclear signal intensity was calculated by assessing the mean pixel intensity within the nuclear mask, which was applied from the DAPI channel, with 2 pixels subtracted from the perimeter of the mask (to ensure that only nuclear signal was assessed). The mean cytoplasmic RNA–FISH signal intensity was calculated by assessing the mean signal intensity in a 30 pixel width ring surrounding each nucleus again with 2 pixels subtracted from the perimeter of the mask to ensure only cytoplasmic signal was measured.

### Immunofluorescence combined with RNA–FISH

Immunofluorescence staining was performed as described above, albeit following this coverslips were not mounted, but instead re-fixed in 4% paraformaldehyde in PBS for 10 min at 4°C. Next RNA–FISH was carried out as previously described (RNA–FISH). All the steps were performed using RNAse inhibitor (Roche) to avoid RNA degradation.

## RESULTS

### THRAP3 promotes resistance to DNA damage and is required for efficient DNA repair

**Figure 1.**
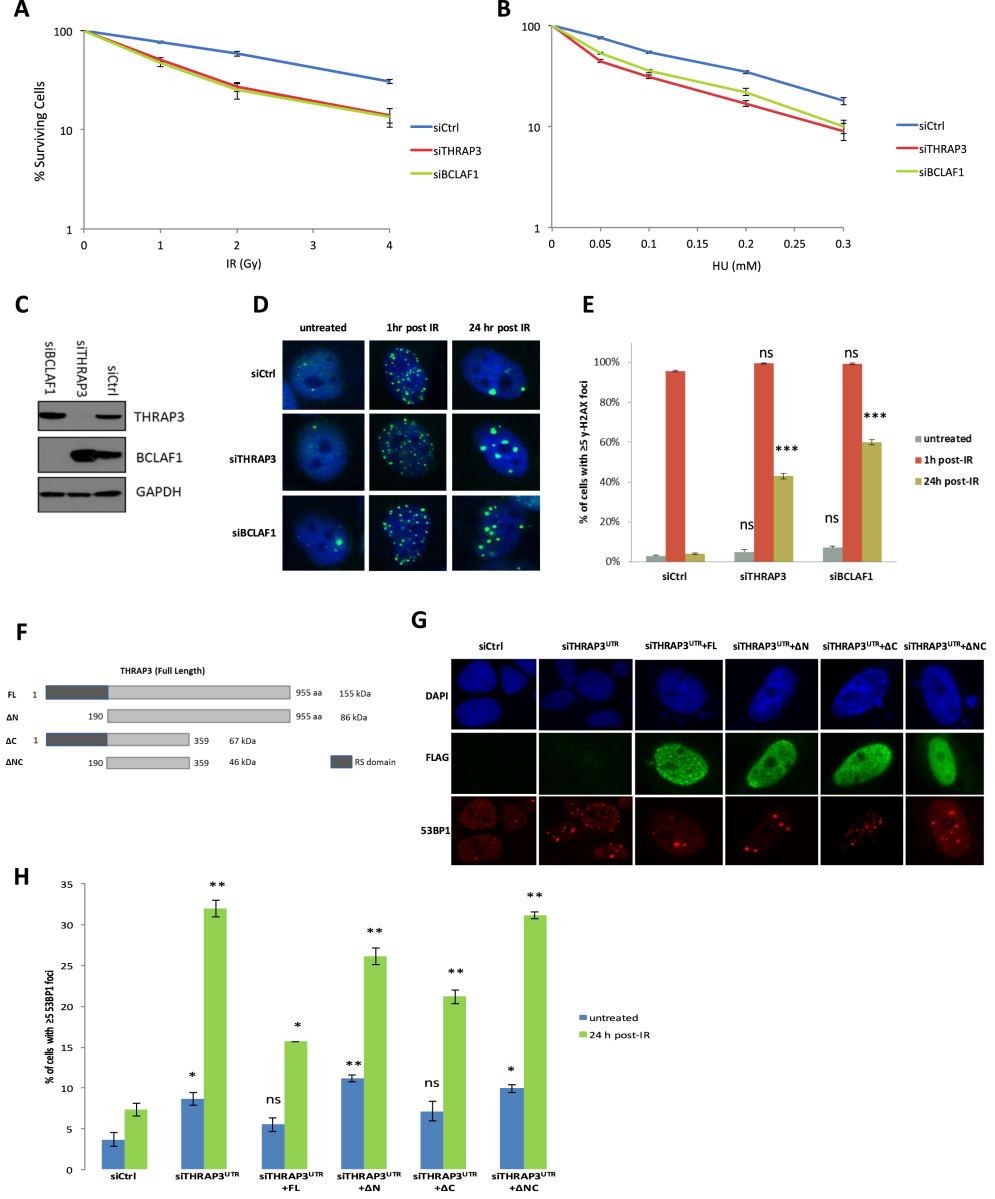
THRAP3 mediates resistance to DNA damage and is required for efficient DNA repair and cellular survival. (**A** and **B**) Clonogenic survival assays demonstrating that transient depletion of THRAP3 induces sensitivity to IR (A) and HU (B) in U2OS cells. Transient knockdown of BCLAF1 (siBCLAF1) was used as a positive control. Mean surviving fraction of three independent experiments is plotted ±SEM. (**C**) Representative western blot demonstrating efficient THRAP3 and BCLAF1 knockdown in cells used for experiments in (A–E). (**D**) Representative immunofluorescent staining of γ-H2AX marked DNA damage in untreated U2OS cells transiently depleted of either BCLAF1 or THRAP3 at 1 and 24 h following 2Gy IR. (**E**) Quantification of three independent experiments described above (≥200 cells were scored/experiment). Mean proportion of cells containing ≥5 γ-H2AX foci is plotted ±SEM. Significant differences in the fraction of cells containing ≥5 γ-H2AX foci were assessed using Student’s two-tailed *t-*test and are indicated by ^∗∗∗^*P* < 0.0005. (**F**) Schematic representation of FLAG-tagged THRAP3 constructs expressing truncated proteins used in immunofluorescent experiments. (**G**) Representative immunofluorescent staining of 53BP1 marked DNA damage in U2OS cells depleted of THRAP3 (using a UTR targeted siRNA) with further transfection of full length and truncated THRAP3 24 h following 2Gy IR. (**H**) Quantification of three independent experiments described above (≥100 cells were scored/experiment). Mean proportion of cells containing ≥5 53BP1 foci is plotted ± SEM. Significant differences in the proportion of cells containing ≥5 53BP1 foci were assessed using Student’s two-tailed *t*-test and are indicated by ^∗^*P* < 0.05, ^∗∗^*P* < 0.005.

As THRAP3 forms a part of the BRCA1/BCLAF1 complex we set out to evaluate the role of THRAP3 in the DDR in the context of BRCA1 mRNA splicing complex function (14,15,22). Similar to BCLAF1, THRAP3 depletion resulted in cellular sensitization to both double strand breaks (IR) and stalled replication forks (HU), thus demonstrating a role for THRAP3 in the maintenance of cellular survival following DNA damage and suggesting an important role within the DDR pathway (Figure 1A–C). To evaluate if THRAP3 may play a role in DNA repair, we assessed DNA repair kinetics in both BCLAF1- and THRAP3-depleted cells, 0, 1 and 24 h following DNA damage. Like BCLAF1 knockdown cells, THRAP3 depleted cells also exhibited a significant defect in their ability to resolve γ-H2AX-marked DNA breaks 24 h after IR treatment, which was rescued by ectopic expression of full length THRAP3 (Figure 1C–H) (14). Interestingly, deletion of either terminus of THRAP3, the N-terminus containing its RNA-binding arginine–serine (RS) domain and the C-terminus of THRAP3 containing no known domains, lead to partial restoration of cellular ability to repair IR-induced DNA damage (Figure 1F–H). In contrast, the central portion of THRAP3 alone (ΔNC) was unable to rescue this DNA repair defect, suggesting that both termini contribute to THRAP3s ability to mediate efficient DNA repair. Moreover, given that deletion of the N-terminal RNA-binding domain of THRAP3 had a weaker effect in restoring the ability of cells to repair DNA damage, it is possible that THRAP3 is involved in the process of DNA repair predominantly through its RNA-binding function (Figure 1F–H).

It is well known that ‘classic’ DNA repair proteins like BRCA1, are directly recruited to the sites of DNA damage where they actively participate in the DNA repair process. In contrast, it has been shown that similar to BCLAF1 and Pinin (members of the SNARP complex), THRAP3 is eliminated from DNA break sites induced by laser micro-irradiation, suggesting that THRAP3 does not play a direct role in DNA repair (14,15,22,23). Such exclusion might be a reflection of transcriptional inhibition, thus resulting in spatial suppression of RNA synthesis and the subsequent absence of RNA-binding proteins at DNA break sites (14,15,22). However, similar proteins, such as the nuclear RNA-binding proteins hnRNPUL 1 and 2, which also appear to be excluded from DNA break sites, have actually been shown to be initially recruited to the sites of DNA damage very rapidly (within 2–3 min), where they play a role in DNA DSB end resection. This rapid and transient recruitment of these proteins to DSBs is better visualized when transcription is globally inhibited (24). Therefore, to examine the possibility of the same RNA-independent localization effect for THRAP3 to DNA breaks, we treated cells with the RNA polymerase II inhibitor 5,6-dichloro-1-β-D-ribofuranosylbenzimidazole (DRB), followed with IR to induce DNA damage (Supplementary Figure S1A). Inhibition of transcription with DRB treatment was confirmed by EU staining (Supplementary Figure S1B). This was carried out at numerous time-points following IR (15 s, 30 s, 1 min, 2 min, 5 min, 10 min, 30 min and 1 h), however, we did not observe any THRAP3 recruitment to DNA break sites, even in the presence of DRB (Supplementary Figure S1A). This suggests that like BCLAF1, THRAP3 plays an indirect role in DNA repair and may instead have a function linking RNA processing/splicing and the DDR. It should be noted that transcriptional inhibition with DRB induces large nuclear THRAP3 foci that do not overlap with DNA repair foci/break sites marked by γ-H2AX (Supplementary Figure S1A). These are reminiscent of stalled transcription factories and are often seen with mRNA processing factors at early time points following transcriptional inhibition.

### Double knockdown of both THRAP3 and BCLAF1 exacerbates cellular sensitivity to DNA damage and DNA repair deficiency

**Figure 2.**
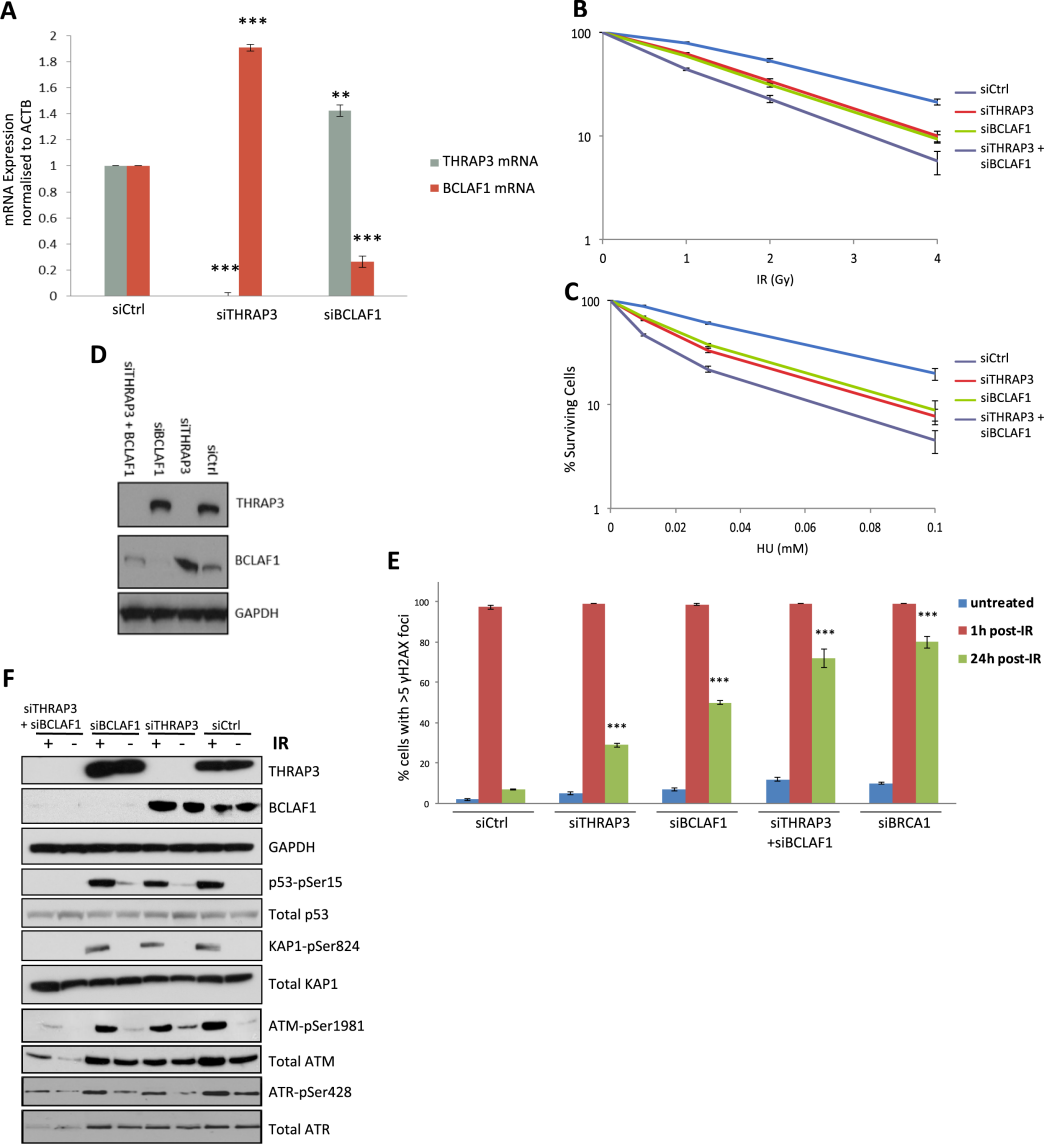
THRAP3 and BCLAF1 functionally compensate for each other. (**A**) THRAP3 and BCLAF1 reciprocal compensation at mRNA level. THRAP3 and BCLAF1 mRNA levels were assessed by quantitative RT-PCR in control (siCtrl) and THRAP3- or BCLAF1-depleted cells and normalized to *actin* (*ACTB*) mRNA. Graphs represent the mean mRNA from three independent experiments ±SEM. Significance of changes was assessed using Student’s two-tailed *t*-test with significant changes indicated by ^∗∗^*P* < 0.005; ^∗∗∗^*P* < 0.0005. (**B** and **C**) Clonogenic survival assays demonstrating that transient depletion of both THRAP3 and BCLAF1 induces severe sensitivity to IR (B) and HU (C) in U2OS cells. Mean surviving fraction of three independent experiments is plotted ±SEM. (**D**) Representative western blot demonstrating transient depletion of THRAP3, BCLAF1 and THRAP3/BCLAF1 in U2OS cells used for DNA repair and clonogenic assays. (**E**) Quantification of three independent experiments of immunofluorescent staining of γ-H2AX marked DNA damage in untreated U2OS cells depleted of either THRAP3, BCLAF1 or THRAP3/BCLAF1, 1 and 24 h following 2Gy IR. Mean fraction of cells containing ≥5 γ-H2AX foci is plotted ±SEM. Significant differences in the fraction of cells containing ≥5 γ-H2AX foci were assessed using Student’s two-tailed *t*-test and are indicated by ^∗∗∗^*P* < 0.0005. (**F**) Representative western blots demonstrating loss of activation of the main downstream members of the signaling cascade such as p53, KAP1 as result of deprivation of ATM and ATR proteins in control (siCtrl) and THRAP3, BCLAF1 and double depleted U2OS cells following 6 Gy IR.

Interestingly, when performing experiments with knockdown of either THRAP3 or BCLAF1, we have observed that depletion of either of them leads to upregulated protein levels of the other (Figure 1C) (25). The same compensation in expression was also detected at the mRNA level using qRT-PCR analysis (Figure 2A). Considering that THRAP3 and BCLAF1 share 39% identity and 66% similarity in their primary sequences, it is perhaps not surprising that they are able to compensate for each other at the mRNA and protein levels.

To further evaluate whether THRAP3 and BCLAF1 are able to functionally compensate for each other within the DDR, we assessed the effect of double depletion of THRAP3 and BCLAF1 on cellular survival and DNA repair following DNA damage. As expected, dual knockdown of both splicing factors led to exacerbated sensitivity of cells to IR and HU treatment compared to depletion of either protein alone (Figure 2B–D). This was also observed at the DNA repair level, with the depletion of both proteins resulting in a repair defect comparable to that seen in BRCA1 depleted cells (Figure 2E) (14). Additionally, IR-treated cells depleted of THRAP3, BCLAF1 or both, displayed multiple complex chromosome aberrations in comparison to control cells, indicating that loss of THRAP3 and BCLAF1 results in increased genomic instability following DNA damage (Supplementary Figure S2A and B). THRAP3/BCLAF1 co-depleted cells did not display more complex aberrations when compared to cells depleted of THRAP3 or BCLAF1 alone. However, a large proportion of THRAP3/BCLAF1 co-depleted cell metaphase spreads were unable to be scored due to hyperfragmentation of and/or absent chromosomes, an indication that we have reached the limit of detection of complex chromosome aberrations using this assay.

The cellular response to DNA damage is controlled by two main kinase signaling cascades, the ATM-Chk2 and ATR-Chk1 pathways, which are activated by double- and single-strand breaks respectively, thus leading to the regulation/activation of multiple cellular processes including DNA repair, apoptosis, transcription, cell cycle checkpoint activation and RNA processing. Thereby, loss of members of this signaling pathway can result in deregulation of DDR pathways resulting in genomic instability and/or cell death. To further investigate the severe sensitivity to DNA damaging agents observed in cells depleted of both THRAP3 and BCLAF1, we assessed the activation of the canonical DDR signaling cascade within these cells. We first examined the activation of various mediator/effector proteins, including p53 and KAP1, by assessing their phosphorylation by ATM/ATR following DNA damage (Figure 2F). Interestingly, double knockdown of THRAP3 and BCLAF1 led to loss of phosphorylation of both p53 and KAP1 following IR (Figure 2F). Following this, we examined the activation of the main upstream kinases ATM and ATR, which are responsible for p53 and KAP1 phosphorylation following DNA damage. Intriguingly, both ATM^Ser1981^ phosphorylation and ATR^Ser428^ phosphorylation, were strongly downregulated in the absence of THRAP3 and BCLAF1 following IR treatment. However, this may be explained by the dramatic downregulation of total expression of the ATM and ATR proteins observed in these cells (Figure 2F). It is worth noting that depletion of either BCLAF1 or THRAP3 alone appeared to have a mild effect on both ATM and ATR expression, suggesting that depletion of either has some effects on ATM/ATR expression, which may be compensated for by upregulation of the reciprocal protein. Interestingly, the same effect i.e. loss of ATM and ATR expression was observed, even in unperturbed cells depleted of THRAP3 and BCLAF1, thus indicating a DNA damage and BRCA1 independent role for THRAP3 and BCLAF1 in the RNA/protein synthesis/maturation process of ATM and ATR (Figure 2F and Supplementary Figure S2C). Importantly, a similar repair defect to that observed in THRAP3/BCLAF1 double depleted cells is observed upon ATM or ATR inhibition (Supplementary Figure S2E and F). Sensitivity to both IR and HU is highly dependent on the status of the cell cycle, as are the levels of a number of DDR proteins such as ATR. Therefore, we assessed cell cycle status in THRAP3 and BCLAF1 depleted cells (Supplementary Figure S2D). Importantly, depletion of THRAP3, BCLAF1 or both did not affect cell cycle distribution in these cells, suggesting that the sensitivity of THRAP3 and BCLAF1 depleted cells to IR and HU is independent of the cell cycle.

### THRAP3 and BCLAF1 are involved in the maturation and further export of transcripts encoding the ATM kinase

The generation and maintenance of a mature functional protein requires the coordinated progression of multiple synthesis steps such as transcription, mRNA-splicing and export of mature mRNA from the nucleus to the cytoplasm for translation into protein. Additionally, protein fidelity is further maintained via regulated degradation at the end of the functional proteins life. Thus, failure at any of these maturation/degradation stages can result in protein loss or dysfunction. As THRAP3 and BCLAF1 double knockdown leads to severely downregulated levels of the ATM protein, we next assessed their possible role in each of the protein’s biosynthesis steps.

To examine whether ATM degradation is affected in THRAP3/BCLAF1 depleted cells, we inhibited protein degradation in THRAP3 and BCLAF1 single- and double knockdown cells using the proteasome inhibitor MG132 (Supplementary Figure S3A). Interestingly, the levels of ATM protein did not change in any of these cells following treatment with MG132, suggesting that dual depletion of THRAP3 and BCLAF1 does not lead to increased ATM protein turnover and its decreased expression level is due to dysfunction of other upstream ATM synthesis steps.

**Figure 3.**
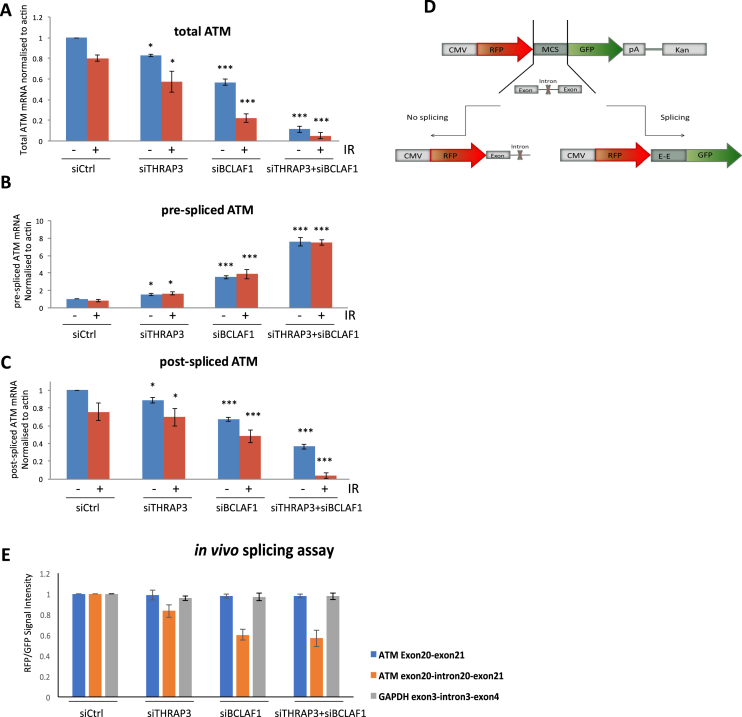
Depletion of THRAP3 and BCLAF1 results in deficient processing of transcripts encoding the ATM kinase. (**A**) Expression level of total post-spliced *ATM* mRNA in control (siCtrl) and THRAP3 (siTHRAP3), BCLAF1 (siBCLAF1) and double depleted cells (siTHRAP3/siBCLAF1). Primers were designed within exons 20–21 spanning intron 20. mRNA expression was assessed via qRT-PCR on cDNA generated from DNAse treated RNA samples and normalized to *ACTB* mRNA levels in the same sample. Graphs represent the mean normalized expression from three independent experiments ±SEM. Significance of changes was assessed using Student’s two-tailed *t-*test with significant changes indicated by ^∗∗^*P* < 0.005; ^∗∗∗^*P* < 0.0005. (**B** and **C**) Nuclear expression levels of pre-spliced (exon 20–intron 20) (B) and post-spliced (exon 20–exon 21) (C) *ATM* mRNAs in nuclear extracts from control (siCtrl), THRAP3 (siTHRAP3), BCLAF1 (siBCLAF1) and double depleted (siTHRAP3/siBCLAF1) cells. mRNA expression was assessed via qRT-PCR on cDNA generated from DNAse treated RNA samples and normalized to *ACTB* mRNA levels in the same sample. Graphs represent the mean normalized expression from three independent experiments ±SEM. Significance of changes was assessed using Student’s two-tailed *t*-test with significant changes indicated by ^∗^*P* < 0.05; ^∗∗^*P* < 0.005; ^∗∗∗^*P* < 0.0005. (**D**) Schematic representation of an mRNA splicing reporter construct containing either an exon–intron–exon sequence from *ATM* (exon 20–exon 21) flanked either side by two fluorescent tags, RFP and GFP or an exon–intron–exon sequence from GAPDH (exon 3–exon 4). The intronic sequence between ATM exons 20 and 21 (intron 20) and GAPDH exons 3 and 4 (intron 3) contain in-frame stop codons, which when unspliced will result in truncation of the encoded protein and expression of RFP but not GFP from this construct. However, efficient *ATM*/GAPDH splicing will result in expression of both RFP and GFP from their respective constructs. A construct containing spliced ATM exon20–21 was also used as a positive control. (**E**) Quantification of splicing reporter assays using *ATM* and *GAPDH* splicing reporter constructs described above in control (siCtrl) and THRAP3, BCLAF1 and double knockdown 293T cells. A total of 10 000 cells were scored per experiment. Graphs represent the mean GFP/RFP signal ratio from three independent experiments ±SEM. Significance of changes was assessed using Student’s two-tailed *t*-test with significant changes indicated by ^∗∗^*P* < 0.005; ^∗∗∗^*P* < 0.0005.

Considering our previous data showing a role for BCLAF1 in the splicing of DDR related transcripts such as *ATRIP, BACH1* and *EXO1*, we next examined the levels of total, post-spliced and pre-spliced *ATM* mRNA in THRAP3 and BCLAF1 single- and double-depleted cells in both unperturbed cells and following DNA damage (14) (Figure 3A–C and Supplementary Figure S2D–M). This revealed that depletion of either THRAP3, BCLAF1 or both, leads to decreased production of the total levels of mature spliced *ATM* mRNA within these cells which occurs irrespective of DNA damage (Figure 3A and Supplementary Figure S4). Moreover, qRT-PCR analysis with exon–intron targeted primers revealed a marked accumulation of pre-spliced *ATM* transcript within the nucleus of THRAP3 and BCLAF1 double depleted cells, which is again independent of DNA damage (Figure 3B). In contrast, we observed a significant decrease in the production of the post-spliced *ATM* transcript in the nucleus of these cells, confirming that efficient splicing of *ATM* requires both THRAP3 and BCLAF1 (Figure 3C and Supplementary Figure S3B). This was observed for all exon/intron junctions assessed (Supplementary Figure S2D–M). Efficiency of RNA fractionation in these experiments was confirmed by examining the levels of the nuclear RNA, *MALAT1* (Supplementary Figure S3B). Interestingly, BCLAF1 depletion was shown to have a more obvious effect on *ATM* splicing than THRAP3, suggesting a more dominant role for BCLAF1 in this process (Figure 3B and C).

To further verify this, we generated an mRNA splicing reporter construct containing an exon–intron–exon sequence from *ATM* (exon20–exon21) flanked either side by two fluorescent tags, RFP and GFP. Importantly, the intronic sequence between exons 20 and 21 contains an in-frame stop codon, which, if left unspliced, will result in truncation of the encoded protein and expression of RFP but not GFP from this construct (Figure 3D). As such, the ratio of GFP/RFP expression can be used to assess ATM exon20–21 splicing efficiency *in vivo*. Based on this, we utilized high-content imaging of cells transfected with this construct to assess GFP/RFP fluorescence intensity. Initially, to assess the functionality of this reporter system, we assessed the GFP/RFP expression ratio in HEK 293T cells transfected with this construct and either mock treated or treated with the splicing inhibitor Spliceostatin A (SSA) (26). As expected, the GFP/RFP signal ratio i.e. splicing efficiency was diminished following SSA treatment (Supplementary Figure S3C). Using this splicing reporter assay, we found that knockdown of BCLAF1, in contrast to THRAP3, resulted in a marked downregulation of ATM splicing, but not GAPDH splicing, confirming that BCLAF1 plays the predominant role in *ATM* splicing (Figure 3E). Interestingly, in comparison to single depleted cells, double knockdown of THRAP3 and BCLAF1 led to a slight reduction in *ATM* splicing, even when compared to BCLAF1 depletion alone, suggesting that both THRAP3 and BCLAF1 function together and/or within the same pathway to regulate *ATM* mRNA splicing (Figure 3C and E).

**Figure 4.**
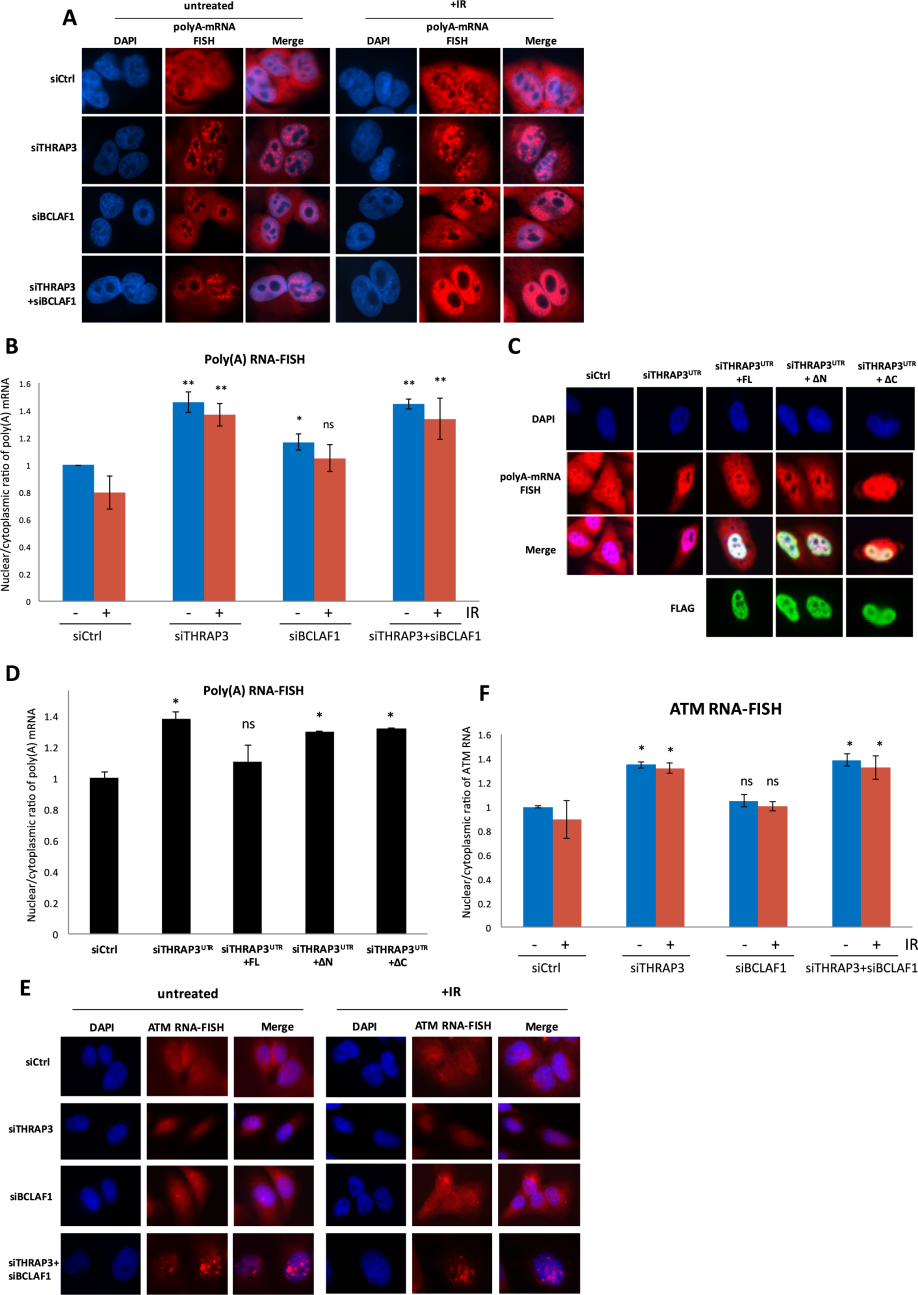
THRAP3 and BCLAF1 are involved in nuclear export of ATM transcripts. (**A**) Representative polyA RNA–FISH showing nuclear accumulation of poly(A) RNA in THRAP3, BCLAF1 and double depleted U2OS cells 72 h post-transfection with appropriate siRNA in the absence and following treatment with IR (6 Gy, 1 h). (**B**) Quantification of nuclear/cytoplasmic ratio of poly(A) RNA fluorescent signal in control, and THRAP3, BCLAF1 and double depleted cells using the described scoring algorithm of three independent experiments. Significant differences in the ratios were assessed using Student’s two-tailed *t*-test and are indicated by ^∗^*P* < 0.05; ^∗∗^*P* < 0.001. (**C**) Representative immunofluorescence followed by poly(A) RNA–FISH in THRAP3 depleted U2OS cells transfected with THRAP3-FL, ΔN and ΔC constructs 72 h post-transfection. (**D**) Quantification of nuclear/cytoplasmic ratio of poly(A) RNA fluorescent signal in control and THRAP3 depleted cells transfected with different truncated constructs using scoring algorithm of three independent experiments. Significant differences in the ratios were assessed using Student’s two-tailed *t*-test and are indicated by ^∗^*P* < 0.05; ^∗∗^*P* < 0.005. (**E**) Representative RNA–FISH with the *ATM* specific probe showing nuclear accumulation of *ATM* mRNA in THRAP3, BCLAF1 and double depleted U2OS cells in the absence and following treatment with IR (6 Gy, 1 h). (**F**) Quantification of nuclear/cytoplasmic ratio of fluorescent signal in (E) was performed using scoring algorithm in three independent experiments. Significant differences in the ratios were assessed using Student’s two-tailed *t*-test and are indicated by ^∗^*P* < 0.05.

Previous studies have suggested that THRAP3 may play a role in mRNA export, a process tightly and sequentially linked with mRNA splicing and required for the stability of mature mRNAs (25). Given that we observed a slight decrease in *ATM* splicing in THRAP3 depleted cells, as well as additional downregulation of *ATM* splicing followed by THRAP3 co-depletion with BCLAF1, we next examined the impact of THRAP3 knockdown on the nuclear-cytoplasmic distribution of poly(A) mRNA and more specifically *ATM* mRNAs, using RNA–FISH with an oligo(dT) or *ATM* specific probes (Figure 4A and B). The nuclear/cytoplasmic ratio of poly(A) mRNA fluorescent signal was measured using an image analysis algorithm designed to quantify the mean nuclear and cytoplasmic pixel intensity (Supplementary Figure S5A). In control siRNA-treated cells, the poly(A) RNA signal was equally distributed between the nucleus and cytoplasm. In contrast, THRAP3 depletion caused the poly(A) RNA species to accumulate in the nucleus, with a concomitant reduction in the cytoplasm (Figure 4A and B). Moreover, export of the poly(A) RNA pool was rescued by ectopic expression of full length THRAP3, suggesting that THRAP3 is required for the efficient transport of mRNAs from the nucleus to the cytosol (Figure 4C and D). Interestingly, expression of either THRAP3 truncation construct, lacking either the N- or C-terminus (ΔN and ΔC) was unable to rescue the nuclear accumulation of poly(A) mRNAs, albeit both ΔN and ΔC THRAP3 appeared to have a partial impact on mRNA export (Figure 4C and D), consistent with their ability to partially rescue DNA repair (Figure 1G and H). Taken together this emphasises the importance of both THRAP3 termini, i.e. the RNA-binding domain and the N-terminus, which shares some homology with BCLAF1, in the process of RNA export. Loss of BCLAF1 also impacts poly(A) RNA export, but to a far lesser extent than THRAP3 (Figure 4A and B). However, given the role of BCLAF1 in the splicing of DDR related transcripts, a pre-requisite for mRNA export, this is perhaps not surprising. In keeping with this, double depletion of THRAP3 and BCLAF1 does not lead to increased nuclear retention of the poly(A) RNA pool, in comparison to THRAP3 depletion alone, thus indicating a predominant role for THRAP3 in the export of BCLAF1/THRAP3 co-regulated targets and suggesting the deficient splicing and export of a large range of transcripts in these cells (Figure 4A and B).

More specifically, depletion of THRAP3 caused nuclear retention of *ATM* transcripts, suggesting that THRAP3 does indeed promote *ATM* mRNA export (Figure 4E and F). Furthermore, the accumulation of *ATM* mRNA observed in THRAP3 deficient cells is not due to increased decay of *ATM* transcripts in these cells (Supplementary Figure S5B). Moreover, the absence of both THRAP3 and BCLAF1 resulted in nuclear accumulation of *ATM* mRNA in nuclear speckles reminiscent of pre-mRNA processing and splicing factories, thus confirming the defective processing of these transcripts in these cells (Figure 4E and F). Taken together, these data suggest that BCLAF1 has a dominant role in the splicing of *ATM*, whereas THRAP3 is predominantly responsible for its export from the nucleus to the cytoplasm.

### THRAP3 and BCLAF1 double knockdown leads to impaired splicing and export of DDR transcripts

Although it is clear that THRAP3 and BCLAF1 coordinate the processing of *ATM* transcripts, it is also clear from our poly(A) RNA FISH experiments that loss of THRAP3 and/or BCLAF1 has a more profound effect on a larger pool of poly-adenylated RNAs. Therefore, in order to identify additional transcripts regulated by THRAP3 and BCLAF1 we performed micro-array based gene expression analysis of nuclear and cytoplasmic RNA pools in THRAP3 and BCLAF1 co-depleted cells. Because mRNA splicing and export are linked processes, we reasoned that cytoplasmic mRNA expression would be affected by loss of both of these steps. First, the efficiency of RNA fractionation was confirmed by examining the levels of the nuclear RNA, *MALAT1* (Supplementary Figure S6A). Following this, cytoplasmic and nuclear RNA pools were hybridized to human whole-transcriptome micro-arrays. Three replicates were performed, allowing the identification of genes with significant changes in expression following THRAP3, BCLAF1 and THRAP3/BCLAF1 depletion (false discovery rate (FDR)-adjusted *P*-value < 0.01). To identify genes whose splicing/export was regulated by THRAP3 and BCLAF1 we filtered for genes downregulated in the cytoplasmic fraction by more than 1.5-fold (Supplementary Figure S6B). In THRAP3 depleted cells 849 genes were downregulated, versus 749 genes downregulated in BCLAF1 depleted cells. Of these genes, only 191 (13.6%) were downregulated in both THRAP3 and BCLAF1 depleted cells (Supplementary Figure S6C). Functional annotation of these genes using DAVID (v6.7) found only small groups of genes involved in multiple cellular pathways including DNA-binding/chromatin structure, cellular glycoproteins, membrane signalling proteins, cytoskeleton structure, etc. In contrast, the abundance of 2538 cytoplasmic RNAs (9.6% of the 26 501 examined) were significantly reduced in the double depleted samples (*P* < 0.05), suggesting a degree of selectivity in how THRAP3 and BCLAF1 regulate mRNA splicing and export. Intriguingly, only 84 of these genes overlapped with those regulated by both THRAP3 or BCLAF1 alone (Supplementary Figure S6D). Functional annotation of these 84 genes revealed that the vast majority encode DNA-binding proteins involved in chromatin structure, including a number of histone variants as well as a small number of DNA-binding transcription factors. However, we reasoned that, due to their ability to functionally compensate for each other, targets of BCLAF1 and THRAP3 mediated splicing and export, such as ATM, are likely to be most noticeably downregulated when both proteins are absent, i.e. the 2538 genes specifically downregulated in THRAP3 and BCLAF1 double depleted cells (Supplementary Figure S6D). Indeed, functional annotation of genes downregulated in the cytoplasmic fraction of THRAP3/BCLAF1 co-depleted cells using DAVID (v6.7) revealed that the largest three gene ontology groups regulated by THRAP3/BCLAF1 are genes involved in transcriptional regulation (385 genes), cell cycle regulation/cell division (241 genes) and DNA Damage/DNA Repair (127 genes). The finding that 385 genes involved in transcriptional regulation were downregulated in THRAP3/BCLAF1 double depleted cells suggests that downregulation of these genes themselves may have secondary/downstream effects on their own target genes. However, given that THRAP3/BCLAF1 have only been transiently depleted for a short period of time (in these cells using siRNA, it is unclear to what extent these secondary effects may impact these results). Nonetheless, and perhaps most strikingly, mRNAs encoding the vast majority of the proteins involved in the Fanconi Anaemia (FA) pathway and essential HR factors were downregulated in the cytoplasmic pool of THRAP3 and BCLAF1 depleted cells, potentially explaining the severe DNA repair defect observed in these cells.

**Figure 5.**
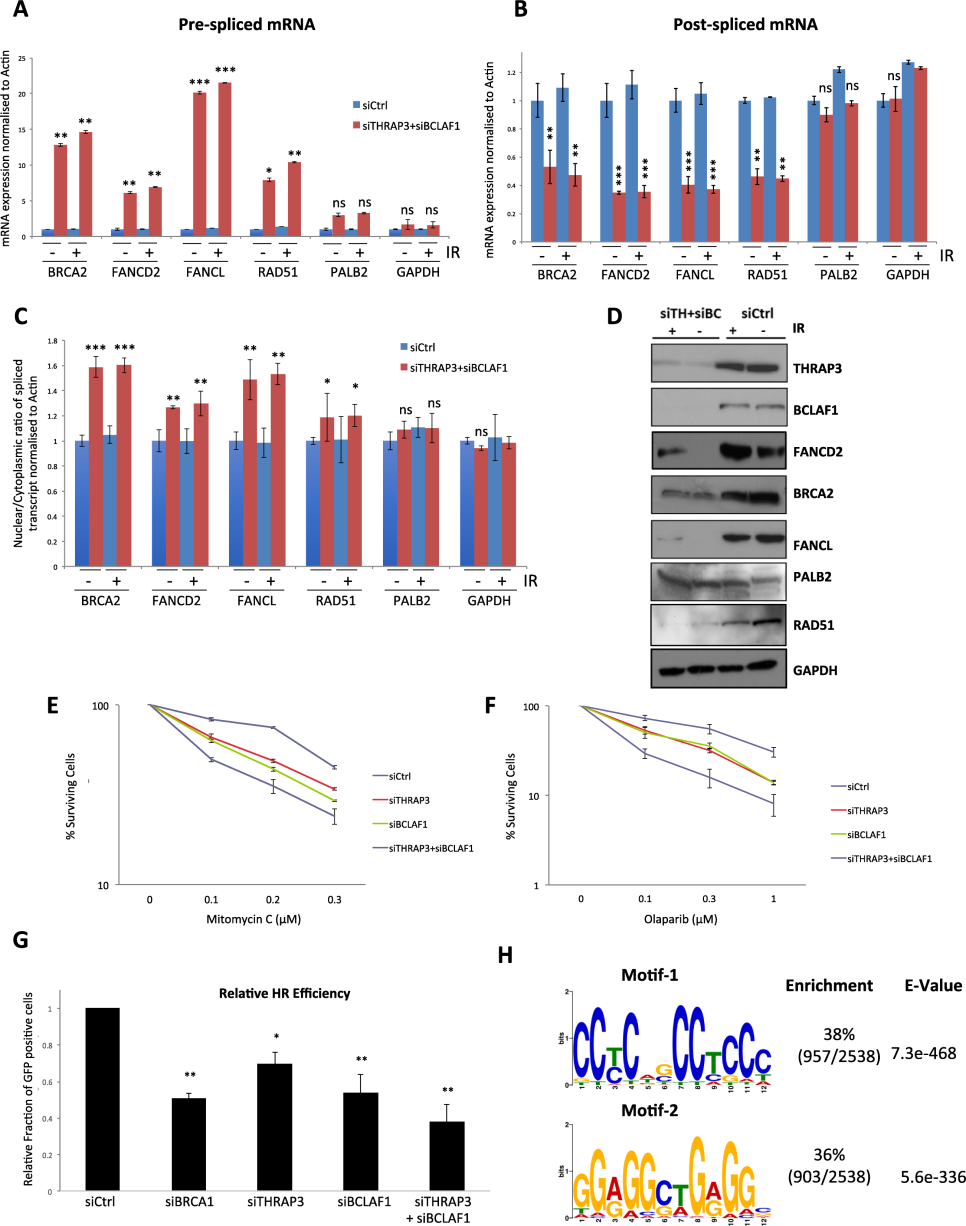
THRAP3 and BCLAF1 double knockdown leads to impaired splicing and export of DDR transcripts. (**A** and **B**) Expression levels of (A) pre-spliced *BRCA2* (*Exon 18–Intron 18*), *FANCD2* (*Exon 36–Intron 36*), *FANCL* (*Exon6–Intron 6*), *RAD51* (*Exon 6–Intron 6*), *PALB2* (*Exon 9–Intron 9*) and *GAPDH* (Exon 3–Intron 3) mRNAs and (B) post-spliced *BRCA2* (*Exon 18-Exon 19*), *FANCD2* (*Exon 36-Exon 37), FANCL* (*Exon 6-Exon 7*), *RAD51* (*Exon 6-Exon 7*), *PALB2* (*Exon 9-Exon 10*) and *GAPDH* (Exon 3–Exon 4) mRNAs. Expression was assessed via qRT-PCR on cDNA generated from DNAse treated nuclear RNA extracts and normalized to ACTB levels in the same sample. Graphs represent the mean of three independent experiments ±SEM. Significance of changes was assessed using Student’s two-tailed *t-*test with significant changes indicated by ^∗∗^*P* < 0.005; ^∗∗∗^*P* < 0.0005. (**C**) Nuclear/cytoplasmic ratio of *FANCD2, FANCL, RAD51, PALB2* and *GAPDH* mRNA. Expression was assessed via qRT-PCR on cDNA generated from DNAse treated RNA samples and normalized to ACTB. Efficiency of nuclear and cytoplasmic RNA extracts was confirmed via qRT-PCR for MALAT1. Graphs represent the mean of three independent experiments ±SEM. Significant differences in the ratios were assessed using Student’s two-tailed *t-*test and are indicated by ^∗^*P* < 0.05; ^∗∗^*P* < 0.005; ^∗∗∗^*P* < 0.0005. (**D**) Representative western blots demonstrating reduced protein expression levels of BRCA2, FANCD2, FANCL and RAD51 in control (siCtrl) and THRAP3/BCLAF1 double depleted cells either untreated or treated with IR (6 Gy 1 h). Protein levels of PALB2 and GAPDH were used as a positive control. (**E** and **F**) Clonogenic survival assays in control (siCtrl), THRAP3, BCLAF1 and double depleted 293T cells following treatment with mitomycin C (MMC) (E) and Olaparib (F). Mean cell viability of three independent experiments is plotted ±SEM. (**G**) Quantification of the DR-GFP reporter assay in control (siCtrl) and BRCA1, THRAP3, BCLAF1, double knockdown U2OS cells. 10 000 cells were scored per experiment using high-content imaging. Graphs represent the mean GFP signal from three independent experiments ±SEM. Significance of changes was assessed using Student’s two-tailed *t-*test with significant changes indicated by ^∗^*P* < 0.05, ^∗∗^*P* < 0.001. (**H**) Overrepresented motifs of lengths 8–20 nt from the 3′ UTR regions of transcripts downregulated in the cytoplasm of THRAP3/BCLAF1 double depleted cells were determined using Multiple Em (expectation maximization) for Motif Elicitation (MEME). Two overrepresented 12-mer motifs in the 3′ UTR of the transcripts identified are indicated.

These findings prompted us to examine the effect of THRAP3 and BCLAF1 depletion on the processing and nuclear export of key downregulated transcripts encoding FA and HR proteins. Indeed, qRT-PCR analysis of pre-spliced RNAs from THRAP3 and BCLAF1 depleted cells shows that unspliced transcripts encoding FANCD2, BRCA2, FANCL and RAD51 accumulate in these cells (Figure 5A and Supplementary Figure S7A). In contrast, the level of post-spliced transcripts encoding these genes is remarkably reduced, thus indicating the importance of BCLAF1 and THRAP3 in the processing of these transcripts (Figure 5B and Supplementary Figure S7B). Expression of pre- and post-spliced *PALB2* (a core HR gene indicated as unchanged in the micro-array data) and *GAPDH* mRNAs, used as negative controls, remained unaltered (Figure 5A and B; Supplementary Figure S7A and B), thus revealing the selectivity of BCLAF1 and THRAP3 for specific target genes. Moreover, *FANCL, BRCA2, FANCD2* and *RAD51* mRNAs detected by qRT-PCR accumulate in the nucleus following THRAP3/BCLAF1 depletion, in contrast to *PALB2* and *GAPDH* mRNAs, whose nuclear/cytoplasmic distribution is unchanged (Figure 5C). Consistent with this, the reduced splicing and export of BRCA2, RAD51, FANCD2 and FANCL observed in these cells results in their downregulated protein expression in the absence of THRAP3 and BCLAF1 in both untreated and IR treated cells (Figure 5D). As expected, the levels of PALB2 and GAPDH proteins remain unaltered (Figure 5D).

To functionally assess the effect of THRAP3 and BCLAF1 depletion on the FA/HR pathways, we examined the ability of THRAP3/BCLAF1 knockdown cells to form FANCD2 foci, a hallmark of a functional FA pathway, following MMC treatment (Supplementary Figure S7C and D). As predicted, double knockdown cells displayed a failure to form FANCD2 foci, thus contributing to the severe sensitivity of double depleted cells to DNA damage.

The interplay between FA and HR pathways in DNA repair is well characterized. Where FA cells are generally sensitive to inter-strand crosslink (ICL)-inducing agents such as cisplatin and MMC they are not sensitive to poly(ADP-ribose) polymerase (PARP) inhibitors, which specifically target HR defective cells. In contrast, cells with mutations in key HR genes e.g. *FANCD1/BRCA2, FANCN/PALB2, FANCR/RAD51* and *FANCS/BRCA1* are notably hypersensitive to PARP inhibitors (27,28). Given that depletion of THRAP3 and BCLAF1 leads to loss of both core FA genes and HR genes, we hypothesised that cells depleted of BCLAF1 and THRAP3 would be sensitive to both ICL-inducing agents and PARP inhibition. Indeed, depletion of BCLAF1 and THRAP3 induced a marked sensitivity to both MMC (an ICL-inducing agent) and the PARPi Olaparib (Figure 5E and F).

To further assess HR efficiency in THRAP3/BCLAF1 depleted cells, we directly assessed HR using a GFP, HR reporter cell line. This cell line harbors the commonly used DR-GFP reporter cassette, which contains two mutant versions of the GFP gene; one with two internal stop codons and an internal I-Sce I restriction site (SceGFP), the other with both 3′ and 5′ truncations (iGFP). Neither SceGFP nor iGFP encode a functional GFP protein; however, following induction of a DSB at the I-Sce I restriction site, HR mediated repair leads to a gene conversion event resulting in the generation of a functional GFP gene. As a result, nuclear GFP expression represents a quantifiable readout of HR function. Using this reporter, single depletion of THRAP3 or BCLAF1 led to a marked decrease in GFP protein expression (Figure 5G). As observed before, depletion of both THRAP3 and BCLAF1 further exacerbated this HR deficiency, suggesting that both processing factors are essential for HR and that again these factors functionally compensate for each other to promote the processing of HR promoting transcripts (Figure 5G). Interestingly, the effect of THRAP3/BCLAF1 co-depletion is similar to loss of BRCA1, a key HR protein directly involved in DSB resection, emphasizing the importance of processing/export of DDR related transcripts by THRAP3/BCLAF1 (Figure 5G).

Our findings collectively suggest a mechanism whereby the THRAP3 and BCLAF1 proteins work at the level of mRNA splicing and nuclear mRNA export to selectively control the expression of a subset of DDR transcripts, thereby promoting efficient HR mediated repair and repair of DNA ICLs. Intriguingly, recent work by Wickramasinghe *et al.* has shown that ALY, an RNA-binding component of the THO/TREX complex that couples RNA transcription to processing and mRNA export, regulates the nuclear export of a similar but distinct pool of transcripts involved in the maintenance of genomic stability (29). This study showed that ALY promotes the nuclear export of these transcripts in response to phosphatidy-linositol (3-5)-trisphosphate (PIP_3_) production, regulated by the inositol polyphosphate multikinase (IPMK). Additionally, this study found that recognition and export of ALY target transcripts is mediated through the binding of ALY to a conserved GAGGCTGGGG RNA-binding sequence within the 3′ untranslated region (UTR) of target transcripts. Additionally, a recent study examining the RNA-binding motif within ALY found that it does not bind RNAs through its conserved RNA recognition motif (RRM) but instead binds transcripts through a conserved WxHD motif found within its N-terminus. Intriguingly, this study found the same conserved RNA-binding motif within both THRAP3 and BCLAF1, suggesting that they may regulate similar target genes and/or bind similar RNA-binding sequences within target transcripts. To test this, we performed MEME (v.4.11.2) analysis of the 3′ UTR of all 2538 genes identified as THRAP3/BCLAF1 target genes through our microarray analysis, to identify over-represented motifs of 6–12 nt in length. This identified two distinct significantly over-represented degenerate 12-mer motifs within the 3′ UTRs of these transcripts (Figure 5H). Wickramasinghe *et al.* identified two highly similar motifs within ALY regulated target genes, however, only the GAGGCTGGGG motif was found to bind ALY. Given that motif-2 identified within our BCLAF1/THRAP3 target genes represents a highly identical binding sequence, GGAGGCTGGGGC and given that both THRAP3 and ALY contain the same conserved RNA-binding motif, it is likely that THRAP3 also recognizes its target transcripts through binding to this sequence. It is possible that THRAP3 and ALY interact to form part of the same RNA-binding/regulatory complex, however, we were unable to detect an interaction between THRAP3 and ALY suggesting that they indeed regulate this overlapping group of transcripts independently (Supplementary Figure S7E).

### THRAP3 cancer associated mutations lead to defective DNA repair and export of THRAP3 target transcripts

Evidence that the BRCA1/BCLAF1 splicing complex may carry out an essential tumor suppressor function arises from the identification of cancer driver mutations within members of the complex, such as BRCA1, SF3B1, U2AF1/2, etc. We therefore set out to assess the impact of the two most common cancer associated mutations within THRAP3, C301>T (R101*) and C2509>T (R837*) (identified through the catalog of somatic mutations in cancer (cancer.sanger.ac.uk)) on THRAP3 function (30). The C301>T mutation results in the introduction of a stop codon thereby producing a 101 aa protein, possessing only two-third of the THRAP3 RS domain. Similarly, the C2509>T variant also results in the introduction of a stop codon and encodes an 837 aa protein that lacks only the end of the C-terminus, in comparison to the full-length 955 aa THRAP3 protein. Both mutations were found in multiple cancers suggesting that they may function as cancer driver mutations.

**Figure 6.**
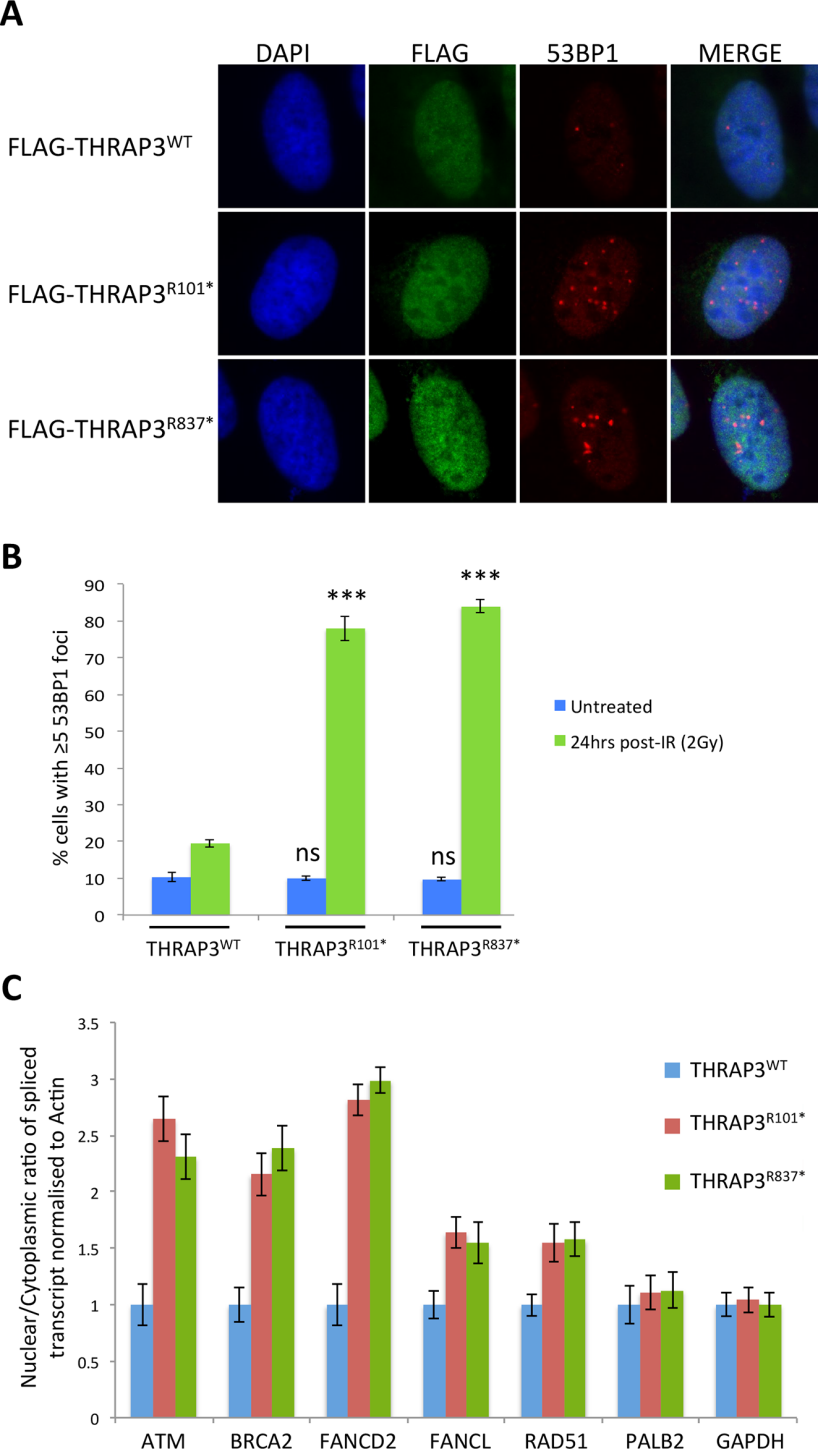
Defect in DNA damage repair and RNA export in THRAP3 associated mutants. (**A**) Representative immunofluorescent staining of 53BP1 marked DNA damage in U2OS cells depleted of THRAP3 with further transfection of THRAP3 cancer associated mutants 24 h following 2Gy IR. (**B**) Quantification of three independent experiments described above (≥100 cells were scored/experiment). Mean fraction of cells containing ≥5 53BP1 foci is plotted ±SEM. Significant differences in the fraction of cells containing ≥5 53BP1 foci were assessed using Student’s two-tailed *t-*test and are indicated by ^∗∗^*P* < 0.005, ^∗∗∗^*P* < 0.0005. (**C**) Nuclear/cytoplasmic ratio of *ATM, BRCA2, FANCD2, FANCL, RAD51, PALB2* and *GAPDH* mRNA levels in control (siCtrl) and THRAP3 depleted cells as well as in cells expressing THRAP3 cancer associated mutations R101* and R837* was quantified by qRT-PCR in three independent experiments. Graphs represent the mean mRNA expression levels relative to control from three independent experiments ±SEM.

THRAP3 constructs were generated by cloning the human THRAP3 coding region in frame with N-terminal myc and FLAG tags. Using site directed mutagenesis, the aforementioned mutations were then introduced into this construct. To examine the effect of these mutations (R101* and R837*) on cellular DNA repair and their potential role in cancer development, U2OS cells, which express endogenous wild-type (wt)-THRAP3, were transfected with wt or mutant THRAP3 constructs and cellular DNA repair capacity assessed, thus mimicking the heterozygous nature of these mutations in cancer cells (Figure 6A). Both THRAP3 cancer-associated mutations affected cellular ability to repair DNA DSBs 24 h after treatment, in comparison to control cells transfected with wt THRAP3, thus mirroring our previous results, which demonstrated that both the N-terminal RS domain and the C-terminal region of THRAP3 are required for efficient DNA repair (Figure 6A and B). In a similar manner, we also examined the capacity of the R101* and R837* mutant THRAP3 to promote the export of THRAP3-dependent transcripts, such as *ATM, BRCA2, FANCD2, FANCL* and *RAD51*. Importantly, both THRAP3 cancer associated mutants confered an mRNA export defect, similar to that observed in THRAP3-depleted cells, leading to defects in mRNA distribution and its accumulation within the nucleus (Figure 6C). Additionally, the nuclear/cytoplasmic ratios of *PALB2* and *GAPDH* transcripts, which are not exported by THRAP3, remained unaltered upon mutant THRAP3 expression (Figure 6C). Given that mutant THRAP3 was expressed in a wt THRAP3 background, in both of these assays, this data suggest that mutant THRAP3 functions in a dominant negative manor, thus abrogating normal cellular DNA repair capacity and potentially contributing to cancer development.

## DISCUSSION

**Figure 7.**
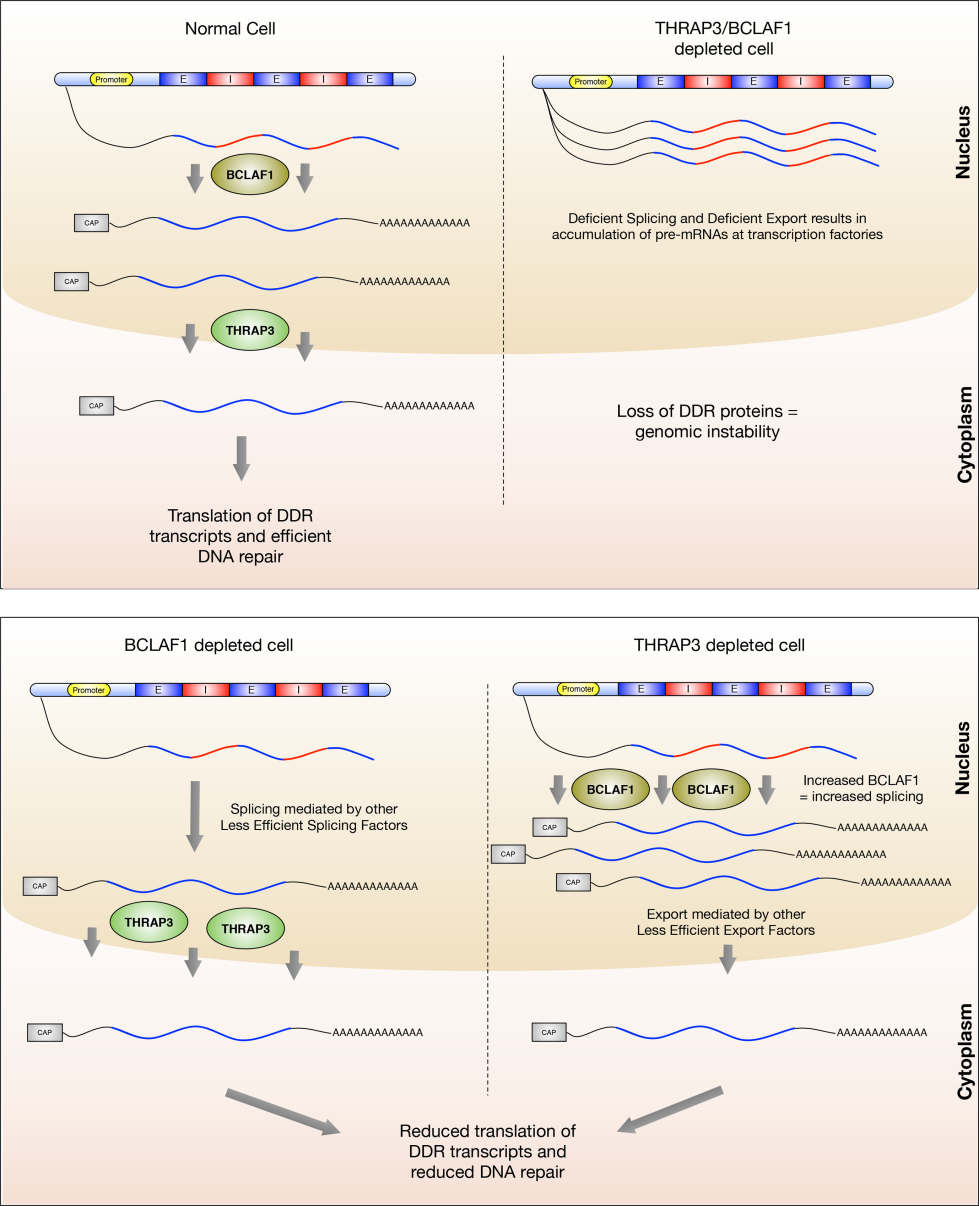
Model of THRAP3 and BCLAF1 function. In normal cells BCLAF1 and THRAP3 function together to promote the selective splicing (BCLAF1) and mRNA export (THRAP3) of a subset of genes, many of which are involved within the cellular DDR pathway. Loss of both BCLAF1 and THRAP3 results in defective splicing and export with subsequent loss of protein expression of THRAP3/BCLAF1 regulated genes. In contrast, in the absence of THRAP3, BCLAF1 is upregulated, which although confers some DDR deficiency, at least partially compensates for THRAP3 loss, presumably via an increased capacity to splice target transcripts which may be exported by alternative, less specific/efficient export proteins. Similarly, loss of BCLAF1 results in upregulation of THRAP3, resulting in increased capacity for export of BCLAF1/THRAP3 target genes, which may be spliced by less specific/efficient splicing proteins, which is able to partially compensate for loss of BCLAF1, resulting in a mild DDR defect.

Recent findings from our group, as well as others, have implicated the RNA processing machinery in the regulation of the cellular DDR. Consistent with this and in addition to their role in the BRCA1/BCLAF1 mRNA splicing complex previously described by our group, here we demonstrate that BCLAF1 and THRAP3 also contribute to the DDR through novel RNA processing functions, which are independent of both DNA damage and BRCA1 (Figure 7). Despite the fact that we did not observe any differential regulation of HR/FA genes regulated by THRAP3/BCLAF1 in response to DNA damage, it is possible that regulation of THRAP3/BCLAF1 following DNA damage e.g. the phosphorylation and/or PARylation events previously reported for these proteins (15,16,31), may fine tune their mRNA splicing/export functions in response to different types and/or levels of genotoxic stimuli.

Within this study, we also found that BCLAF1 and THRAP3 function together to promote the splicing and mRNA export of target genes, with BCLAF1 playing a predominant role in splicing, whereas THRAP3 plays a more dominant role in mRNA export. Despite the seemingly distinct roles in RNA processing, depletion of BCLAF1 does indeed have an impact on mRNA export and *vice versa*, depletion of THRAP3 has a slight impact on mRNA splicing (Figure 7). However, given the tightly linked nature of these two processes, this is perhaps not surprising, as mRNA splicing is required for mRNA export and may indeed result in the formation of export complexes onto spliced transcripts (9,32). Additionally, it is likely that lack of mRNA export i.e. the removal of spliced transcripts from transcription factories, impinges on mRNA splicing. Indeed, a number of biochemical studies have shown that down regulation of mRNA export can directly impact mRNA splicing competence (2). Our study also found that the downregulation of mRNA splicing and export of BCLAF1/THRAP3 target genes in cells depleted of these proteins lead to almost complete loss of their encoded proteins, whereas single depletion of THRAP3 and BCLAF1 had only a mild effect on many of these proteins. In keeping with this, we found that depletion of either THRAP3 or BCLAF1 alone, lead to upregulated transcription and subsequent expression of the other protein e.g. BCLAF1 knockdown lead to increased THRAP3 expression. This suggests that the splicing function of BCLAF1 and the export function of THRAP3 are, at least in part, redundant functions and can in some way be compensated for by upregulation of the reciprocal protein/function. It is possible that BCLAF1 upregulation in the absence of THRAP3 serves to increase the concentration of spliced THRAP3/BCLAF1 target genes thereby saturating mRNA processing factories with these transcripts and facilitating the export of these transcripts via other export proteins/adapters, e.g. ALY. Similarly, the upregulation of THRAP3 in the absence of BCLAF1 may serve to increase the export of THRAP3/BCLAF1 target genes that may be spliced, albeit less efficiently, by other mRNA splicing components. However, more work will be required to understand this complex process, which may require gene/transcript specific models beyond the scope of this study. Additionally, although we have shown that THRAP3 and BCLAF1 compensation occurs at the transcriptional level, the mechanism through which this occurs requires further investigation. It may be that THRAP3 and BCLAF1 co-regulate themselves at the splicing and export level. Alternatively, they may co-regulate the levels of transcription factors that regulate the expression of each other. Indeed, a number of transcription factors e.g. E2F’s, identified as THRAP3/BCLAF1 target genes in this study, do indeed bind the promoters of THRAP3 and BCLAF1 directly (33).

Given that many of the genes regulated by THRAP3/BCLAF1 encode proteins within the HR/ICL repair pathway, it is not surprising that THRAP3 and BCLAF1 depletion lead to decreased HR activity and cellular sensitivity to both the ICL inducing agent mitomycin C and the PARP inhibitor, olaparib, which is known to target HR deficient cells. Intriguingly, as described earlier, the mRNA export of an overlapping but distinct set of genes has been reported to be regulated by ALY, an RNA-binding protein that functions to couple transcriptional processing and mRNA export (29). ALY itself is recruited to mRNP particles during/coincident with splicing, forming at nuclear speckle structures, which are accumulation sites for mRNA maturation factors (8). ALY then recruits the export receptor protein TAP, which mediates the export of mRNAs. THRAP3, also forms nuclear speckles, which are characterized by their increase in size and intensity following transcriptional inhibition (34) (Supplementary Figure S1A) and has also been shown to bind TAP (20), suggesting that it may function in a similar fashion to ALY. Intriguingly, a recent study has found that ALY does not selectively bind transcripts through its RRM, but instead this occurs through two highly conserved WxHD and HDxR motifs (35). These motifs were also found within THRAP3, supporting the hypothesis that THRAP3 may function in a similar fashion to ALY and may indeed bind to, and regulate, similar transcripts (35). In support of this, we also found similar 3′ UTR-binding sequences within THRAP3 regulated transcripts, albeit the motifs we have identified are slightly longer than those identified within ALY target transcripts (ALY recognition motif = 10-mer GAGGCTGGGG versus THRAP3 recognition motifs = 12-mer GGAGGCTGGGGC) (29). Nevertheless, it is clear that THRAP3 and ALY function distinctly to regulate discrete pools of transcripts. Indeed, ALY has been shown to play a more global role in the regulation of a large pool of transcripts, which is regulated by the binding of phosphatidy- linositol (3-5)-trisphosphate (PIP_3_), the levels of which are controlled by IPMK (29). ALY has been shown to directly bind PIP_3_, through a series of conserved arginine and lysine residues within its N-terminus (36), suggesting that PIP_3_ binding may alter ALYs structure, thereby regulating its ability to bind and selectively regulate HR-related transcripts. In contrast, THRAP3 does not appear to contain a similar PIP_3_-binding region/residues and therefore it is unlikely that IPMK/ PIP_3_ regulate its ability to bind to and regulate target transcripts.

Given the role of THRAP3/BCLAF1 in the regulation of DDR proteins, we hypothesized that some cancer-associated mutations within THRAP3, particularly those that result in loss of THRAP3 structure/function, may confer a DNA repair defect on cells harboring these mutations. To examine this we tested the impact of two cancer-associated mutations R101* and R837* on THRAP3 function. Ectopic expression of either THRAP3 mutant, even in a THRAP3 wt background, resulted in downregulated export of key THRAP3/BCLAF1 target genes involved in HR and ICL repair and a subsequent defect in cellular DSB repair, which was similar to that observed in THRAP3 depleted cells. This suggests that THRAP3 truncation mutants function in a dominant negative manner, potentially suggesting that THRAP3 may function within a complex requiring more than one intact THRAP3 molecule and/or that mutant THRAP3 prevents export of target transcripts by wt THRAP3. Taken together, this data suggests that patients with tumors harboring truncating mutations in THRAP3, or other mutations that result in loss of THRAP3 protein expression/function, may respond better to DNA damaging chemotherapies, radiotherapy and targeted therapies, such as PARPi’s, that target tumors with an intrinsic DNA repair defect.

In conclusion, this study highlights the multifunctional nature of the RNA processing factors THRAP3 and BCLAF1 in the selective splicing and export of a large subset of transcripts involved in the cellular DDR. Moreover, as cancer associated mutations within THRAP3 result in deregulated export of target transcripts and a subsequent DNA repair defect, this data suggests that THRAP3/BCLAF1 may function to suppress tumor development and/or act as biomarkers of response to therapy.

## Supplementary Material

Supplementary Data
