# Supplementary material for: The RNA processing factors THRAP3 and BCLAF1 promote the DNA damage response through selective mRNA splicing and nuclear export
Source: Nucleic Acids Res. 2017 Nov 3;45(22):12816–33. doi: 10.1093/nar/gkx1046 (PMC5728405; doi:10.1093/nar/gkx1046)
Supplement: Supplementary Data [file gkx1046_supp.pdf]

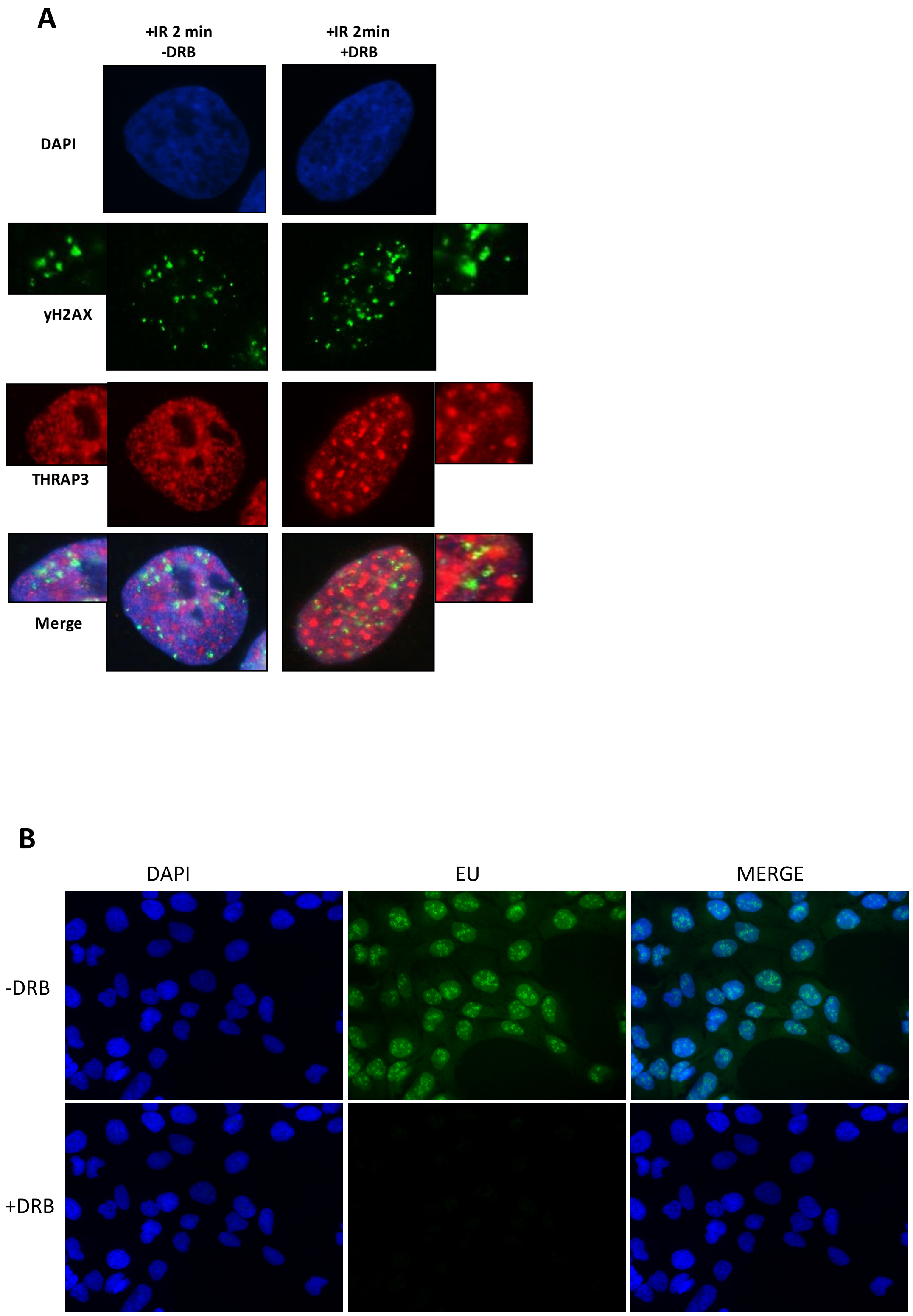

Supplementary Figure 1

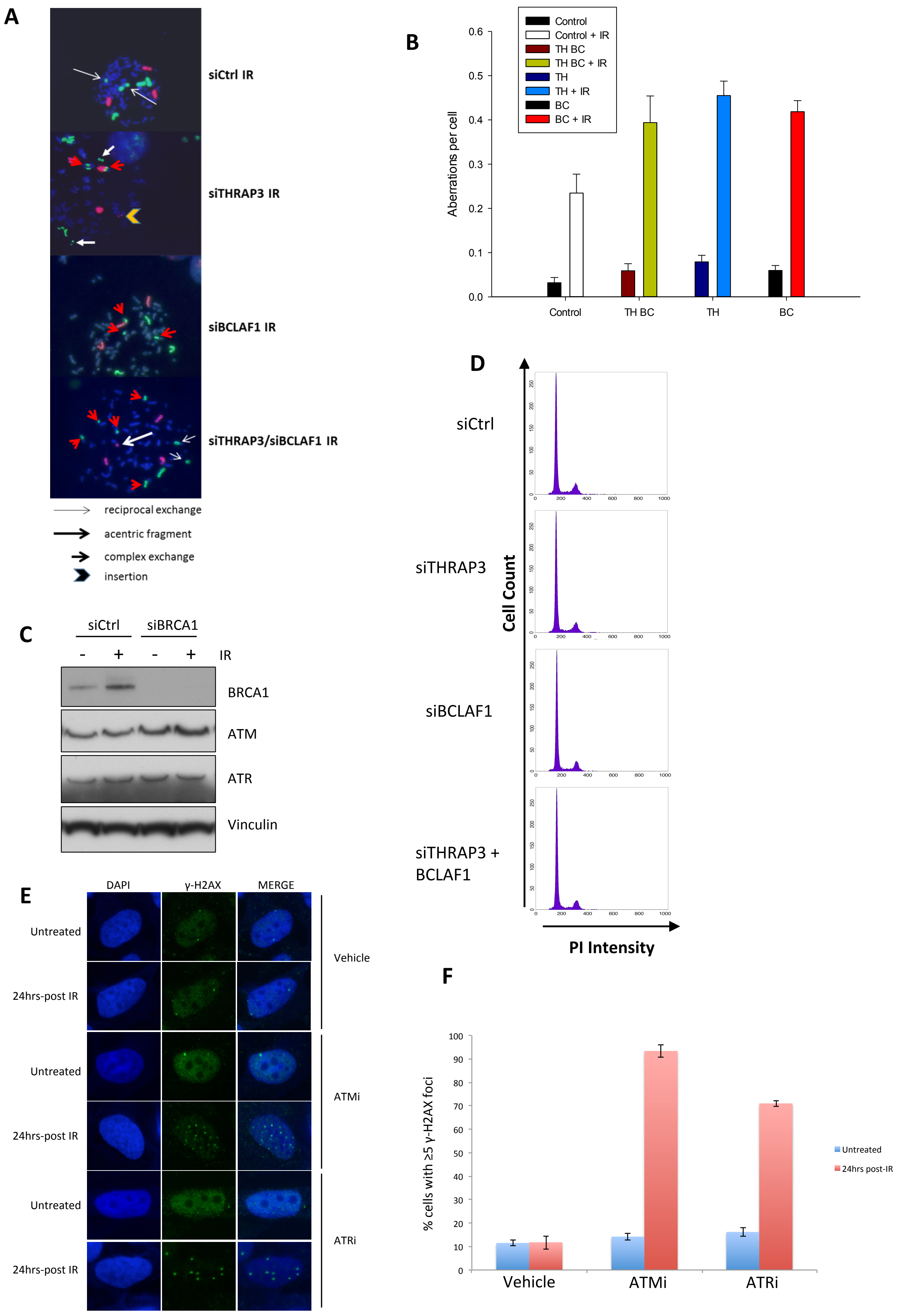

Supplementary Figure 2

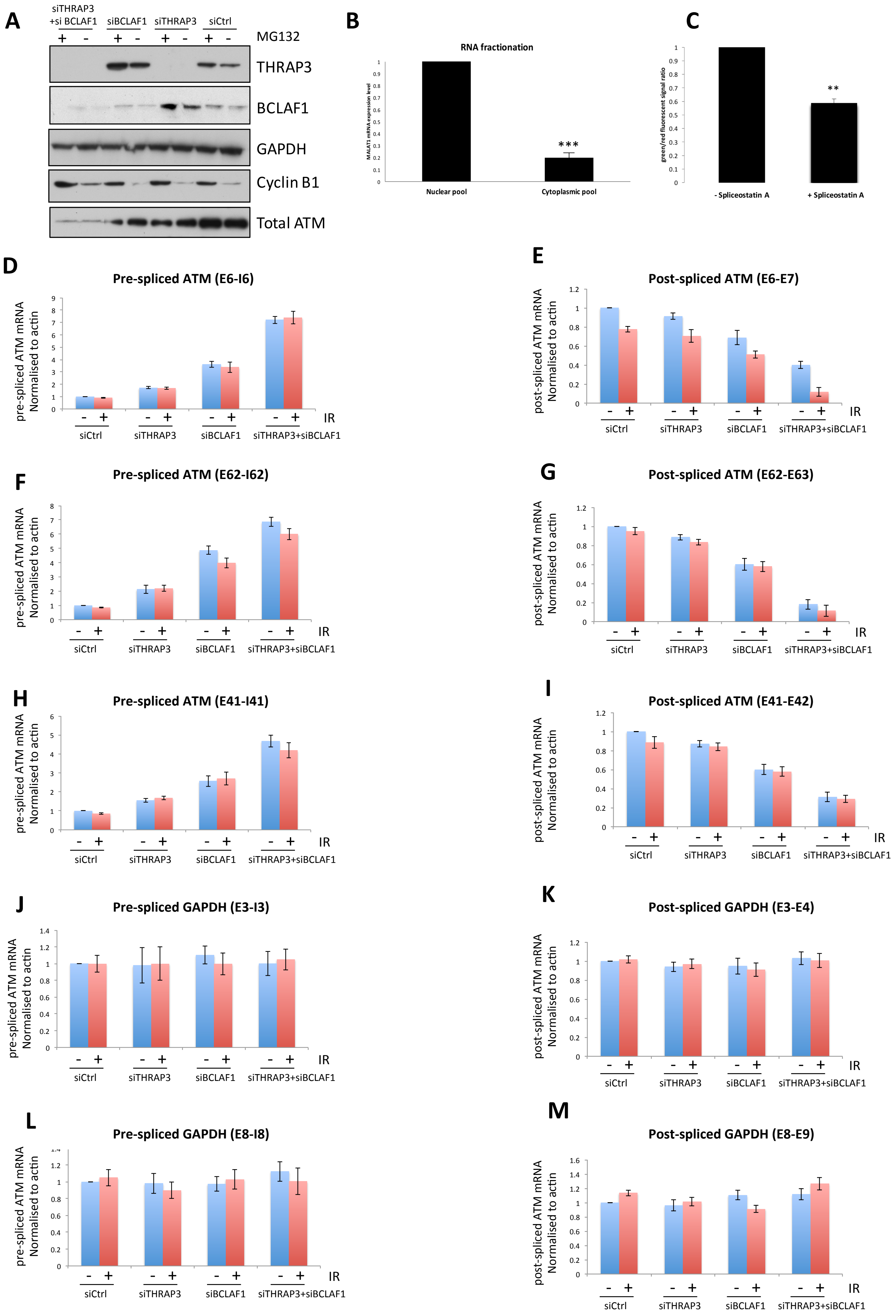

Supplementary Figure 3

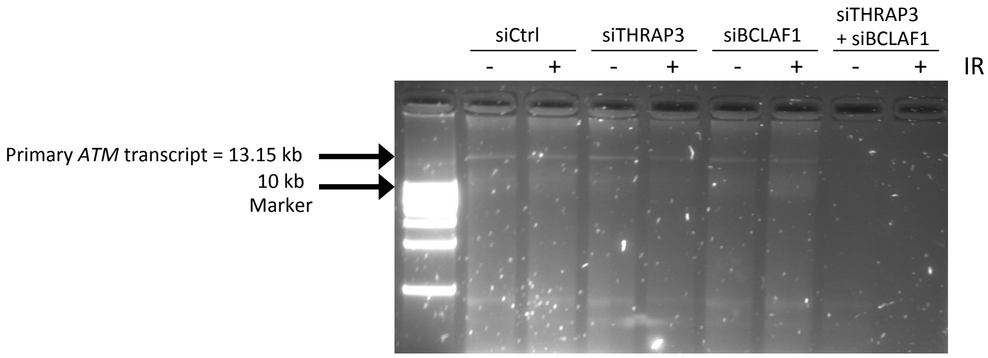

Supplementary Figure 4

A

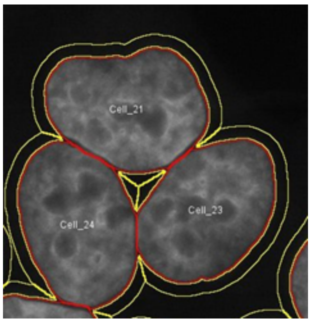

B

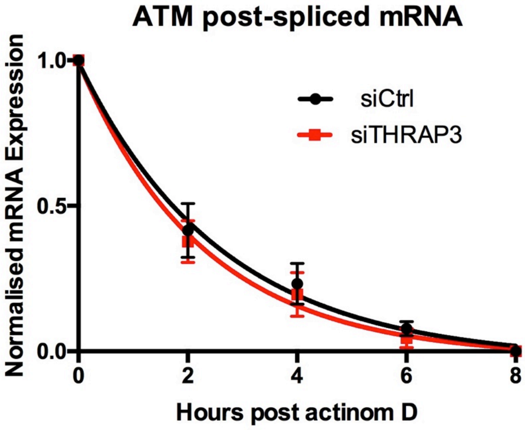

Supplementary Figure 5

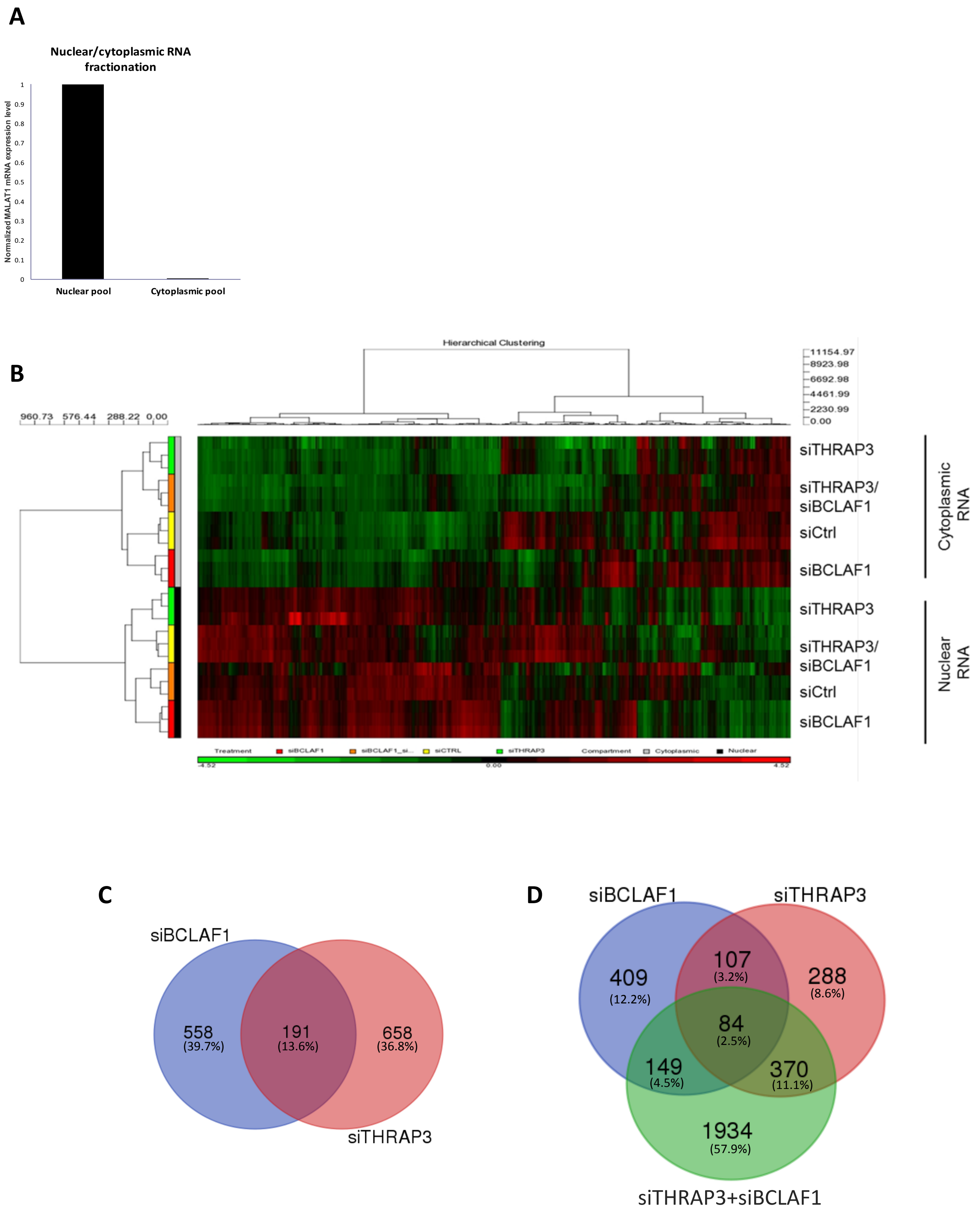

Supplementary figure 6

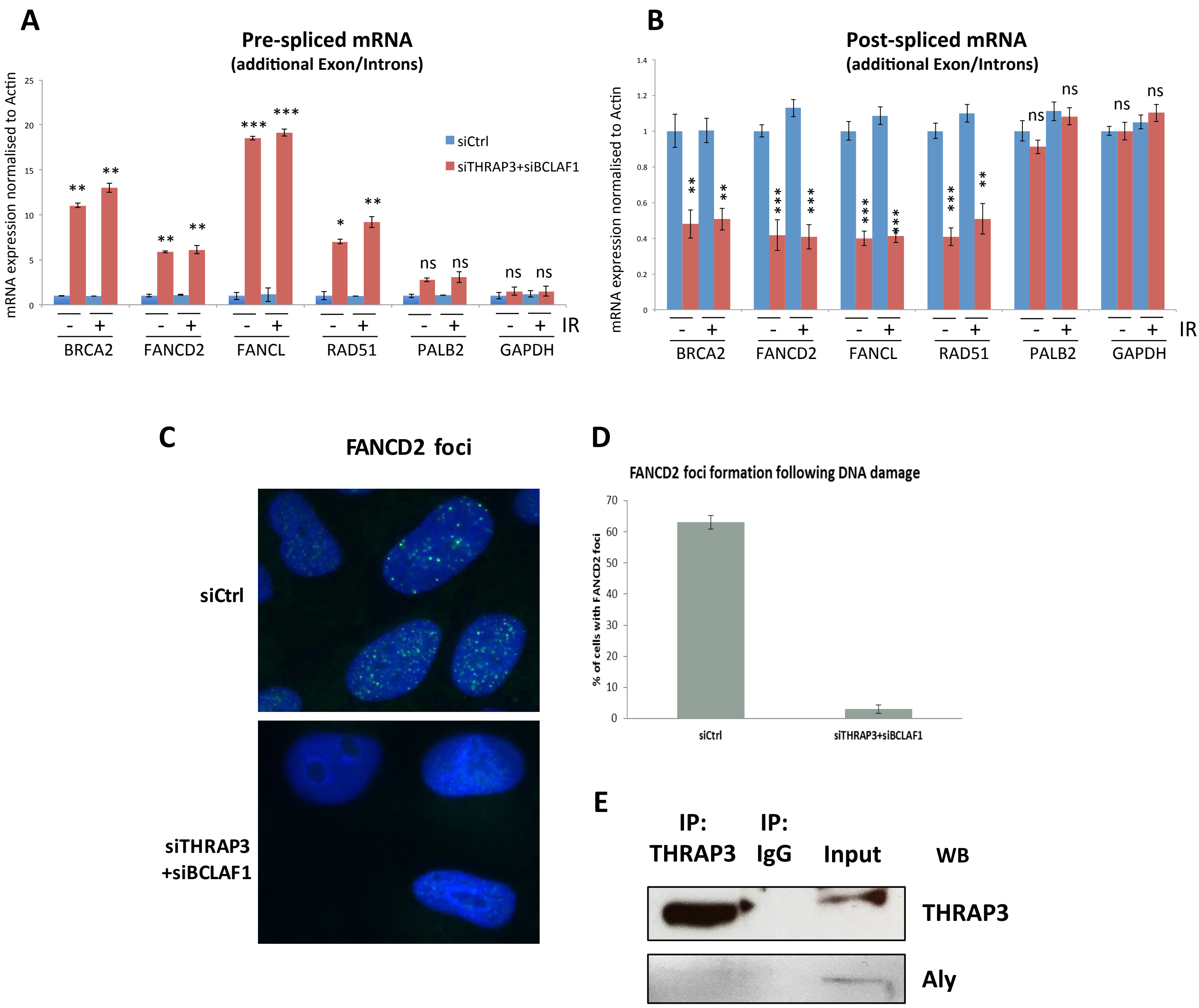

Supplementary figure 7

SUPPLMENTARY FIGURE LEGENDS

Supplementary Figure 1.

A. Representative immunofluorescent staining of γ-H2AX marked DNA damage and THRAP3 protein exclusion from sites of IR-induced damage (2 Gy) 2 min after irradiation in U2OS cells pre-treated with DMSO (-DRB ((5,6 dichloro- ribofuranosylbenzimidazole)) or DRB (100 μM, 2 hours) to inhibit transcription. **B.** Representative image of 5-ethynyl uridine (EU) incorporation, a marker of newly synthesised RNA, in cells treated as above. EU was stained with Alexa-fluor-488 using the Click-iT™ RNA Alexa Fluor™ 488 Imaging Kit (ThermoFisher Scientific). Lack of EU incorporation in DRB treated cells confirms inhibition of transcription in these cells.

Supplementary Figure 2.

A. Representative metaphase spreads of control (siCtrl), THRAP3, BCLAF1, and THRAP3/BCLAF1 depleted 293T cells 24 hr following 2Gy IR. FISH-mediated whole chromosome painting (chromosome 1, green; chromosome 2, red) was used to identify complex chromosome aberrations. Specific aberrations are described in the legend and highlighted in each image. **B**. Quantification of total chromosome aberrations in control, THRAP3, BCLAF1, and double depleted 293T cells 24 hr after mock irradiation or irradiation with 2 Gy IR. Graphs represent the mean number of chromosome aberrations/metaphase from three independent experiments ± SEM (≥50 metaphases scored/experiment). **C**. Representative Western Blots demonstrating no impact of ATM and ATR expression in BRCA1 depleted U2OS cells in either the absence or presence of DNA damage (IR (6Gy, 1hr)). **D.** Representative cell cycle profiles of control, THRAP3, BCLAF1 and THRAP3/BCLAF1 depleted U2OS cells showing no impact of cell cycle progression/status on these cells. **E.** Representative immunofluorescent staining of γ-H2AX marked DNA damage 24 hours following IR-induced damage (2 Gy) in U2OS cells pre-treated with ATM inhibitor (KU60019 (1μM), or ATR inhibitor (AZD6738 (100nM)) for 1 hour prior to irradiation and

remaining in inhibitor over the 24hr repair period. **F.** Quantification of three independent experiments of immunofluorescent staining of γ-H2AX marked DNA damage in depicted in E. Mean fraction of cells containing ≥5 γ-H2AX foci is plotted ± SEM. This data shows that inhibition of either ATM or ATR leads to a significant DSB repair defect in these cells.

Supplementary Figure 3.

A. Representative Western blot demonstrating unaltered ATM protein levels following inhibition of proteasomal mediated protein degradation with MG132 (5 μM, 16 hours) in control (siCtrl) and THRAP3, BCLAF1 and double depleted U2OS cells. **B**. The efficiency of nuclear/cytoplasmic RNA fractionation was confirmed by examining the levels of the nuclear RNA, *MALAT1* in nuclear and cytoplasmic fractions harvested from the same cells by qRT-PCR using primers specific to *MALAT1*.. Results are plotted relative to nuclear RNA levels and show the mean of triplicate experiments ± SEM. **C**. Validation of the splicing reporter assay of the ATM construct described in (**Figure 3D**) using the splicing inhibitor Spliceostatin A (SSA). 293T cells were transfected with the ATM construct and 24 hours later treated with SSA (1 μM, 16 hr). Fluorescent ratio of RFP and GFP signals was measured using high-content imaging. **D-I** Nuclear expression levels of pre- (d, f, h) and post-spliced (e, g, i) *ATM* mRNA in control (siCtrl) and THRAP3 (siTHRAP3), BCLAF1 (siBCLAF1) and double depleted cells (siTHRAP3/siBCLAF1). Primers were designed against the indicated exons (E)/introns (I) with mRNA expression normalized to *ACTB* mRNA. Graphs represent the mean normalized expression from three independent experiments ± SEM. **J-M** Nuclear expression levels of pre- (d, l) and post-spliced (k, m) *GAPDH* mRNA in control (siCtrl) and THRAP3 (siTHRAP3), BCLAF1 (siBCLAF1) and double depleted cells (siTHRAP3/siBCLAF1). Primers were designed against the indicated exons/introns with mRNA expression normalized to *ACTB* mRNA. Graphs represent the mean normalized expression from three independent experiments ± SEM.

Supplementary Figure 4

In order to asses if alternative *ATM* splice variants were produced in response to DNA damage and or THRAP3/BCLAF1 depletion, cDNA generated from mRNA harvested from mock or IR treated 293T cells was used for PCR analysis using a forward primer in exon1 and a reverse primer within exon 63 of ATM. These primer sets encompass the 2 most commonly expressed *ATM* transcripts (ENST00000278616.8 and ENST00000452508.6). In response to DNA damage and/or depletion of THRAP3, BCLAF1 or both THRAP3 and BCLAF1 no alternative ATM transcript variant were detected. Surprisingly, although this was a saturating PCR (40 cycles) no full length spliced ATM transcript was detectable in the THRAP3/BCLAF1 double depleted cells.

Supplementary Figure 5.

A. Representative image demonstrating scoring algorithm applied masks used for measuring nuclear/cytoplasmic ratios of poly(A) mRNA fluorescent signal (see text for further details). This algorithm, was designed to identify cellular nuclei from the DAPI channel of a two channel image (channel 1 being DAPI and channel 2 being our fluorescent RNA-FISH probe signal) and apply this as a mask on the channel-imaged for fluorescent RNA FISH probes. Importantly, image acquisition settings were not changed between conditions or experiments. To assess nuclear RNA-FISH signal intensity, and to ensure optimal separation of the nuclear and cytoplasmic image compartments, the mean nuclear signal intensity was calculated by assessing the mean pixel intensity within the nuclear mask, which was applied from the DAPI channel, with 2 pixels subtracted from the perimeter of the mask (to ensure that only nuclear signal was assessed). The mean cytoplasmic RNA-FISH signal intensity was also calculated by assessing the mean signal intensity in a 30 pixel width ring surrounding each nucleus again using a 2 pixel margin between the nuclear mask and the cytoplasmic ring. **B**. qRT-PCR analysis of post-spliced *ATM* mRNA level following inhibition of transcription with Actinomycin D treatment (10μg/ml) in U2OS cells transfected with control (siCtrl) and THRAP3 siRNAs. Data points represent the mean normalised expression of three independent experiments +/- SEM.

Supplementary Figure 6

A. The efficiency of nuclear/cytoplasmic RNA fractionation for microarray was confirmed by examining the levels of predominantly nuclear retained RNA, MALAT1 in nuclear and cytoplasmic fractions by qRT-PCR using primers specific to MALAT1 RNA. Results are plotted relative to nuclear RNA levels and show the mean of triplicate repeats from one representative experiment ± SEM. **B**. Microarray based gene expression analysis of nuclear and cytoplasmic RNA pools in control (siCtrl) and THRAP3/BCLAF1 co-depleted U2OS cells. Following fractionation cytoplasmic and nuclear RNA pools were hybridized to human whole-genome microarrays. Three replicates were performed and an adjusted p-value obtained (adjusted p value <0.05). Hierarchical clustering was then performed on significantly regulated genes. Downregulated/upregulated transcripts are shown in green and red, respectively. **C.** Venn diagram showing overlap between gene transcripts downregulated in the cytoplasm of siBCLAF1 and siTHRAP3 depleted cells. **D.** Venn diagram showing overlap between gene transcripts downregulated in the cytoplasm of siBCLAF1, siTHRAP3 and siTHRAP3/BCLAF1 double depleted cells.

Supplementary Figure 7.

A-B Expression levels of additional; (A) pre-spliced *BRCA2 (Exon 2-Intron 2), FANCD2 (Exon4-Intron4), FANCL (Exon 11-Intron 11), RAD51 (Exon 2-Intron 2), PALB2 (Exon 9-Intron 9)*, and *GAPDH* (Exon 3-Intron 3) mRNAs, and (B) post-spliced *BRCA2 (Exon2-Exon 3), FANCD2 (Exon 4-Exon 5), FANCL (Exon 11-Exon 12), RAD51 (Exon2-Exon3), PALB2 (Exon1-Exon2)*, and *GAPDH* (Exon 3-Exon 4) mRNAs. Expression was assessed via qRT-PCR on cDNA generated from DNAse treated nuclear RNA extracts and normalized to ACTB levels in the same sample. Graphs represent the mean of three independent experiments +/- SEM. Significance of changes was assessed using Student's two-tailed t test with significant changes indicated by **p < 0.005; ***p < 0.0005. **C**. Representative immunofluorescence

staining of FANCD2 foci in control (siCtrl) and THRAP3/BCLAF1 co-depleted U2OS cells following treatment with 2μM MMC for 6 hours. **D**. Percentage of the cells with FANCD2 foci in (**C**) in control and THRAP3/BCLAF1 knockdown cells in three independent experiments. Significant differences were assessed using Student's two-tailed t test and are indicated by **p < 0.005. **E.** Co-immunoprecipitation assay demonstrating absence of interaction between endogenous THRAP3 and Aly in 293T cells.

SUPPLEMENTAL MATERIALS AND METHODS

Metaphase Spreads and Chromosomal Aberration Analysis

293T cells were transfected with siRNAs (siCtrl, siTHRAP3, siBCLAF1, siTHRAP3 + siBCLAF1) and incubated for 48hrs. Cells were then mock irradiated or irradiated with 2Gy IR using an X-RAD 225 X-ray generator (Precision X-ray Inc. Branford, CT, USA) at a dose rate of 0.591 Gy.min-1. Twenty hours after irradiation cells were treated with Colcemid (0.4μg/mL) for a further 4hrs after which cells were trypsinized and spun down. Metaphases were collected by resuspending pellet with hypotonic 75 mM KCl for 20 min at 37 °C, followed by fixation for 20 min at 4 °C in freshly prepared Carnoy solution (3 : 1 v/v methanol/acetic acid). After two more washes in Carnoy solution, cells were dropped onto pre-warmed wet slides and air-dried at room temperature and aged at room temperature for 7 days. Aged slides were hybridised with whole chromosome fluorescence-labelled DNA probes (XCP, Whole-Chromosome Probe, MetaSystems) directed to chromosomes 1 (fluorochrome FITC) and chromosome 2(fluorochrome Texas Red) as per manufacturer's instructions. DNA denaturation (72 °C for 3 min) and hybridisation (37 °C for 8 hr) were performed using the HYBrite chamber system (Vysis). All chromosomes were counterstained with DAPI (Sigma). Coded slides were viewed with an epifluorescence microscope (Axioplan2 imaging MOT, Carl Zeiss) connected to an automated system (Metafer 4 software, MetaSystems) for slide scanning and three-colour image acquisition. Chromosome aberrations were analysed on stored images. All slides were scored blind by the same scorer. All types of aberrations were scored separately and categorised in simple exchanges (i. e. translocations and dicentrics), either visibly structurally complete or incomplete, acentric excess fragments and complex exchanges. No centromere probe was used but centromeres were clearly distinguishable as bright bands under DAPI illumination.

Cell treatments

Cells were treated with DRB/MG132/Spliceostatin A/actinomycin D – all reagents were purchased from Sigma.

Co-Immunoprecipitations

2μg of primary antibody (THRAP3, Bethyl Laboratories) was coupled to an anti-rabbit Dynabeads (Invitrogen) and added to 2mg of pre-cleared 293T whole cell lysate (ELB Lysis Buffer: 250mM NaCl, 5mM EDTA, 50mM HEPES, 0.1% v/v NP40). Immunoprecipitations were carried out overnight at 4^oC followed by 6 washes with ELB Buffer. Immunoprecipitated proteins were then resuspended in 2x LDS loading buffer (Invitrogen).

qRT-PCR and splicing analysis of additional ATM and GAPDH exons

1μg of DNAse (Invitrogen) treated RNA was used for cDNA synthesis using the Transcriptor High Fidelity cDNA Synthesis Kit (Roche Applied Science) according to the manufacturer's instructions. qRT-PCR was performed using primers specific to each transcript or to *ACTB* mRNA, on both RT positive and RT negative generated cDNA. All reciprocal qRT-PCR reactions performed on RT–ve cDNA were assessed for the absence of any cDNA amplification indicating no genomic DNA contamination prior to further analysis of data. All qRT-PCR reactions were carried out on a ROCHE LightCycler 480 using SYBR Green 480 I Master Mix (ROCHE) as per the manufacturer's instructions. mRNA concentration levels were then assessed using the ROCHE Relative Quantification algorithm, utilising in-run standard curve qRT-PCR data generated for each primer set from a serially diluted RNA standard. For quantitative splicing analysis, introns/exons were chosen for assessment in splicing analysis based on their suitability for optimal qRT-PCR primer design. Primers spanning exon-exon boundaries (post-spliced) and exon-intron boundaries (pre-spliced) were designed using the ROCHE universal probe library and manually inputting exon-exon and exon-intron flanking sequences. Using these primers (listed below) levels of pre-spliced and post-spliced mRNAs were assessed as above and normalised to ACTB expression levels within the same samples. Primers used for qRT-PCR analysis were as follows: MALAT1 Fwd: 5-

GACGGAGGTTGAGATGAAGC -3, MALAT1 Rev: 5- ATTCGGGGCTCTGTAGTCCT -3, ACTB Fwd: 5- AGACCTGTACGCCAACACAG -3, ACTB Rev: 5- GGAGCAATGATCTTGATCTTCA -3, ATM_Exon6_Fwd: 5- TGCTGTTACCAAAGGATGCTG -3, ATM_Exon7_Rev: 5- TGAGGAAGATAGTAAGAGCTGCT -3, ATM_Intron6_Rev: 5- TGAGTCTAAAACATGGTCTTGCA -3, ATM_Exon41_Fwd: 5- CTGTGGTGGAGGGAAGATGT-3, ATM_Exon42_Rev: 5'- CCCACATTGCTTCGTGTTCA -3', ATM_Intron41_Rev: 5- GGGTTTTACACACACATAACTCC -3, ATM_Exon62_Fwd: 5- AGGCCGGAAGATGAAACTGA-3, ATM_Exon63_Rev: 5- CTTGTCCACCAACACTGAGC -3, ATM_Intron62_Rev: 5- TGAGGTGAACAGTTTAAAGGCC -3, GAPDH_Exon8_Fwd: 5- CTGACTTCAACAGCGACACC -3, GAPDH_Exon9_Rev: 5- GTGGTCCAGGGGTCTTACTC -3, GAPDH_Intron8_Rev: 5- AGAGTTGTCAGGGCCCTTTT -3, GAPDH_Exon3_Fwd: 5- TCACCAGGGCTGCTTTTAAC -3, GAPDH_Exon4_Rev: 5- TGACAAGCTTCCCGTTCTCA -3, GAPDH_Intron3_Rev: 5- TACGTGAGGGTATGAAGGGG -3, FANCD2_Exon4_Fwd: 5- GGAGACACCCTTCCTATCCC-3, FANCD2_Intron5_Rev: 5- GCCCACTCAACTTTATACCAGT, FANCD2_Exon5_Rev: 5-GGAGACACCCTTCCTATCCC-3, BRCA2_Exon2_Fwd: 5- AGCATTGGAGGAATATCGTAGGT-3, BRCA2_Intron2_Rev: 5- AGCAACACTGTGACGTACTG-3, BRCA2_Exon3_Rev: 5- AGGGTGGAGCTTCTGAAGAA-3, FANCL_Exon11_Fwd: 5-CATGCTTTCAGGGATCCAGA-3, FANCL_Intron11_Rev: 5- CAGCCCTGAAATCAATCCCC-3, FANCL_Exon12_Rev: 5- TCAGGAATGGTACCGTCAAGT-3, PALB2_Exon1_Fwd: 5-TGCTCTTTTCGTTCTGTCGC-3, PALB2_Intron1_Rev: 5- AGATGATACTGCTGCCCTCG-3, PALB2_Exon2_Rev: 5-ACACGAAAGGACCTGGAAGT-3, Rad51_Exon2_Fwd: 5-CAAATGCAGATACTTCAGTGGAA-3, Rad51_Intron2_Rev: 5- TGGGTCTTGACCTTGGTAGT-3, Rad51_Exon3_Rev: 5-CTCCTTCTTTGGCGCATAGG-3.
